# PGC‐1α Transcriptionally Regulated by ChREBP Mitigates Neuropathic Pain Through Promoting Microglial Fatty Acid Oxidation and Anti‐Inflammatory Response

**DOI:** 10.1002/cns.70744

**Published:** 2026-01-10

**Authors:** Ziwei Hu, Jiahui Pang, Xinli Liu, Yun Zhao, Yi Lu, Hui Chen, Hui Zeng, Youxin Yu, Yubai Zhao, Lijie Gao, Xuefei Zhang, Jian Jin, Kangling Wang, Yu Shi, Hongrui Zhan, Wen Wu

**Affiliations:** ^1^ Center of Rehabilitation Medicine, Zhujiang Hospital Southern Medical University Guangzhou China; ^2^ School of Rehabilitation Sciences Southern Medical University Guangzhou China; ^3^ Guang Dong Engineering Technology Research Center of Brain Function Assessment and Neuroregulation Rehabilitation Guangzhou China; ^4^ Institute of Exercise and Rehabilitation Science, Zhujiang Hospital Southern Medical University Guangzhou China; ^5^ State Key Laboratory of Respiratory Disease, National Clinical Research Center for Respiratory Disease, National Center for Respiratory Medicine, Department of Thoracic Surgery and Oncology, Guangzhou Institute of Respiratory Health The First Affiliated Hospital of Guangzhou Medical University Guangzhou China; ^6^ Department of Clinical and Rehabilitation Medicine Guiyang Healthcare Vocational University Guizhou China; ^7^ Department of Rehabilitation The Fifth Affiliated Hospital of Sun Yat‐Sen University Zhuhai China

**Keywords:** ChREBP, fatty acid oxidation, microglia, neuropathic pain, PGC‐1α

## Abstract

**Background:**

Neuropathic pain (NP), a chronic disorder caused by somatosensory nervous system lesions, severely impairs the quality of life. Microglial metabolic reprogramming and neuroinflammation drive NP progression. Although ChREBP (key metabolic regulator) protects against NP, its specific mechanisms remain unclear.

**Methods:**

NP rat model was established via spared nerve injury (SNI) surgery, and mechanical allodynia was evaluated using Von Frey tests. ChREBP expression in microglia was detected through immunofluorescence, RT‐qPCR, and western blot. Functional studies involved ChREBP knockdown/overexpression to assess effects on microglial polarization, neuroinflammation, neuronal excitability, pain behaviors, and fatty acid metabolism. Mechanisms were explored via dual‐luciferase reporter and chromatin immunoprecipitation assays.

**Results:**

Mechanical pain thresholds were significantly decreased on the ipsilateral side after SNI. ChREBP was upregulated in SDH microglia after SNI and in LPS‐stimulated microglia in vitro. ChREBP knockdown inhibited anti‐inflammatory microglial polarization, exacerbated neuroinflammation, and aggravated pain. Conversely, ChREBP overexpression promoted the anti‐inflammatory phenotype, suppressed neuroinflammation, and alleviated pain. ChREBP enhanced microglial fatty acid oxidation and energy metabolism. Mechanistically, ChREBP bound to the TFBS1 site on the PGC‐1α promoter to activate its transcription. PGC‐1α overexpression rescued the impairments caused by ChREBP knockdown, including reduced fatty acid oxidation, suppressed anti‐inflammatory polarization, elevated inflammatory factors, and increased neuronal excitability. The protective effects of ChREBP were attenuated by the fatty acid oxidation inhibitor Etomoxir.

**Conclusions:**

ChREBP alleviates NP by enhancing microglial fatty acid oxidation and anti‐inflammatory phenotype via PGC‐1α transcriptional activation, revealing a novel metabolic‐immune axis for potential NP therapy.

## Graphical abstract

ChREBP transcriptionally regulates PGC‐1α, which enhances the expression of fatty acid oxidation genes, promotes the fatty acid oxidation process, and thereby increases the polarization of anti‐inflammatory microglia, reduces the level of inflammatory factors, and improves pain symptoms.

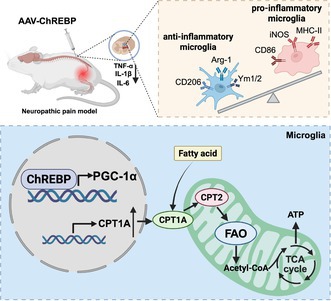

## 1 Introduction

Neuropathic pain (NP) is a complex and chronic disease caused by lesions or defects of the somatosensory nervous system [1]. The features of NP encompass spontaneous pain, hyperalgesia, and mechanical allodynia [2]. Studies indicate that the overall prevalence of NP is 9.2%, with chronic pain significantly impairing daily activities, reducing work productivity, and deteriorating quality of life [3, 4]. Therefore, elucidating the pathological mechanisms of NP and discovering innovative therapeutic targets hold significant scientific value.

The pathogenesis of NP is intricate, involving the entire nociceptive pathway [5]. Microglia, which are resident in the spinal dorsal horn (SDH), rapidly respond to spared nerve injury (SNI) [6, 7]. Under normal physiological conditions, microglia exhibit a relatively quiescent phenotype [8]. However, when immune homeostasis is disrupted, they polarize into two extreme phenotypes: pro‐inflammatory and anti‐inflammatory phenotypes [9]. Interestingly, microglia with different phenotypes exhibit significant changes not only in morphology, cell number, and gene expression levels but also in their metabolic patterns [10, 11]. Pro‐inflammatory microglia rely on aerobic glycolysis, while anti‐inflammatory microglia use fatty acid oxidation to promote oxidative phosphorylation [12]. A recent study has demonstrated that higher levels of fatty acid oxidation in anti‐inflammatory microglia enhance oxidative phosphorylation, providing a sustained energy supply, suppressing inflammation, and promoting tissue repair [13]. Thus, reducing inflammatory responses or enhancing the reparative effects of anti‐inflammatory microglia may contribute to the amelioration of NP.

Carbohydrate‐responsive element‐binding protein (ChREBP) primarily regulates the expression of genes related to glucose and lipid metabolism, thereby maintaining the homeostasis of glucose and lipid metabolism in the body [14, 15]. In recent years, with the deepening of research, the function of ChREBP in the nervous system has been gradually revealed. ChREBP not only participates in the regulation of energy metabolism in nerve cells but also plays an important role in processes such as neuroinflammation and nerve injury repair [16]. In the SNI model, peripheral nerve injury leads to changes in the local nerve microenvironment, including inflammatory cell infiltration, cytokine release, and metabolic disorders [17]. In response to these pathological changes, the protective increase in ChREBP reduces the inflammatory response, thereby alleviating pain and promoting the recovery of nerve function [18]. Given that microglial metabolic reprogramming is critical for NP pathogenesis, and considering ChREBP's dual roles as a metabolic regulator and a participant in pain modulation, it is critical to explore whether ChREBP acts as a pivotal molecule in regulating NP pathogenesis by targeting microglial metabolism.

In this study, we uncovered the specific mechanisms by which ChREBP alleviates pain by promoting microglial fatty acid oxidation, enhancing the proportion of anti‐inflammatory microglia, inhibiting inflammatory responses, and reducing the excitability of spinal neurons. We identified peroxisome proliferator‐activated receptor γ coactivator 1α (PGC‐1α) as an important downstream effector of ChREBP in the regulation of microglial metabolism and NP. This study clarifies the protective role of ChREBP in NP by regulating microglial metabolism and highlights its potential as a novel target for developing metabolism‐based therapeutic strategies for NP.

## 2 Methods

### 2.1 Animals

All experiments were reviewed and approved by the Laboratory Animal Ethics Committee of Zhujiang Hospital, Southern Medical University (Ethics Approval No.: LAEC‐2022‐144) and were performed in strict accordance with the ethical guidelines for laboratory animal use established by the International Association for the Study of Pain (IASP) [19]. Male Sprague–Dawley (SD) rats, aged 8–10 weeks and weighing 200–250 g, were purchased from Zhuhai Bestest Bio‐Tech Co. Ltd. (Zhuhai, China). The rats were housed in a standard specific pathogen‐free (SPF) environment at a temperature of 20°C–26°C, with a 12‐h light/12‐h dark cycle, and were provided with food and water ad libitum.

### 2.2 Construction of the NP Rat Model

As previously reported [20, 21], SNI was performed on SD rats to induce NP. Rats were anesthetized with 2%–3% isoflurane for induction inhalationally and maintained with 1%–1.5% isoflurane. The sciatic nerve was exposed at the mid‐thigh level, and its branches—including the common peroneal nerve, tibial nerve, and sural nerve—were carefully isolated. The common peroneal and tibial nerves were tightly ligated proximally using 4–0 silk sutures, while the sural nerve was left intact. The sham group underwent the same procedure, except that only the sciatic nerve and its three branches were identified, without ligation or transection of any nerves.

### 2.3 Stereotactic Injection of AAV Into the SDH

Spinal cord stereotactic injection was adapted from Perrine's method [22]. Rats were anesthetized with isoflurane, and their dorsal fur was shaved and disinfected. After identifying the T12‐L2 lumbar vertebrae, a laminectomy was performed to fully expose the L4‐L6 spinal cord segments. The spine was stabilized with rods to ensure precise positioning of the microsyringe needle at the midpoint of the L5 segment. The stereotactic instrument coordinates were reset to zero, with final parameters set as *X* = 0.5 mm, *Y* = 0.5 mm, and *Z* = 0.5 mm [18]. Adeno‐associated virus (AAV) constructs or control reagents were administered at a rate of 0.2 μL/min, with a total volume of 1 μL. The needle was retained for 10 min post‐injection to prevent reflux. The AAVs used included those targeting microglia to interfere with ChREBP (AAV9‐F4/80‐sh ChREBP, 5.47 × 10^13^ vg/mL), the AAV encoding ChREBP (AAV9‐F4/80‐oe ChREBP, 8.69 × 10^13^ vg/mL), and the AAV encoding PGC‐1α (AAV9‐F4/80‐oe PGC‐1α, 9.36 × 10^13^ vg/mL), all provided by Shandong Weizhen Biotechnology Co. Ltd. (Jinan, China).

### 2.4 Intrathecal Catheterization

Intraspinal catheterization was performed according to a method reported by Hou et al. [23]. After L5‐L6 intervertebral foramen was exposed, sterile PE‐10 catheter was inserted into the epidural space and gently advanced caudally to the lumbar enlargement of the spinal cord. Correct placement was confirmed by reflexive tail or hindlimb movement, indicating dura penetration. The catheter was tunneled subcutaneously and externalized at the nape of the neck. On the day after surgery, 10 μL of 2% lidocaine was injected through the catheter. Correct placement was confirmed by the occurrence of transient hindlimb paralysis. Etomoxir (HY‐50202A; MedChemExpress, USA) was first dissolved in DMSO and subsequently diluted with 0.9% NaCl. After SNI surgery, either Etomoxir (0.05 mg/kg) or vehicle (0.9% NaCl) was administered daily via the catheter for 14 consecutive days [24, 25].

### 2.5 Behavioral Test

The von Frey (Aesthesio, RWD) test was conducted to evaluate mechanical allodynia in rats of each group, which was used as the primary behavioral measure in this study [26]. Filaments of varying stiffness (ranging from 1.0 g to 60 g) were applied perpendicularly to the plantar surface of the ipsilateral hind paw. The force was gradually increased until the filament bent and then maintained for 2 to 5 s. Responses such as foot withdrawal, licking, or swinging were marked as “X”; no response was recorded as “O”. Once an initial “XO” or “OX” response occurred, four additional stimuli were applied to complete the procedure. Behavioral testing was conducted in a blinded manner. The calculation of the 50% paw withdrawal threshold (PWT) was based on the formula: 10^[Xf+kdelta]^/10,000 [27].

### 2.6 Immunofluorescence Assay

Frozen spinal cord tissues from the L4‐L6 segments were embedded in OCT compound and then cut into 25 μm sections. To determine the localization of ChREBP changes within the SDH, we subdivided the dorsal horn into laminae I–VI according to Rexed's laminar classification [28]. After blocking at room temperature for 1 h, the antibodies were prepared with QuickBlock primary antibody dilution buffer (P0262; Beyotime): goat polyclonal antibody to Iba‐1 (1:250, ab5076; Abcam), mouse monoclonal antibody to GFAP (1:400, 3670S; CST), mouse monoclonal antibody to NeuN (1:400; MAB377, Millipore), rabbit polyclonal antibody to ChREBP (1:200; NB400‐135, Novus Biologicals), mouse monoclonal antibody to PGC‐1α (1:200, 66369‐1‐Ig; Proteintech), rabbit polyclonal antibody to iNOS (1:200, BS1186; Bioworld) and rabbit polyclonal antibody to Arg‐1 (1:200, BS67738; Bioworld), and incubated at 4°C overnight. Secondary antibodies included donkey anti‐rabbit (AF555, A31572; Invitrogen), donkey anti‐goat (AF488, A11055; Invitrogen), donkey anti‐mouse (AF488, A21202; Invitrogen), and donkey anti‐mouse (AF647, A31571; Invitrogen). Secondary antibodies were diluted 1:500 and incubated at room temperature for 1 h. Finally, the coverslips were mounted on slides using an anti‐fading reagent containing DAPI (Solarbio, S2110). Fluorescence images were captured using a confocal laser microscope (Nikon AX) and analyzed using ImageJ software to measure mean fluorescence intensity or the number of positive cells.

### 2.7 Patch‐Clamp Recordings

Spinal cord segments L4‐L6 were isolated from rats, embedded in agarose gel, and sectioned at 300 μm using a vibratome in ice‐cold (4°C) artificial cerebrospinal fluid (ACSF) containing (in mM): 125 NaCl, 2.5 KCl, 2 CaCl_2_,1 MgCl_2_, 26 NaHCO_3_, 1.25 NaH_2_PO_4_, and 10 glucose [29]. Slices were transferred to an incubation chamber continuously bubbled with 95% O_2_/5% CO₂ and allowed to equilibrate for 20 min. Whole‐cell patch‐clamp recordings were performed at room temperature (20°C–25°C) using an EPC 10 USB Patch Clamp Amplifier (HEKA Elektronik, Germany). Spontaneous excitatory postsynaptic currents (sEPSCs) were recorded at a holding potential of −70 mV. Data were digitized at 10 kHz, filtered at 2 kHz, and analyzed using PatchMaster (HEKA) and Clampfit 10.3 (Molecular Devices). Recordings were obtained for a minimum of 5 min per cell for offline analysis.

### 2.8 Cell Culture

An immortalized rat microglial cell line (HAPI) was obtained from Shanghai Enzyme‐linked Biotechnology Co. Ltd. (Shanghai, China) [30]. Cells were cultured in DMEM containing 10% FBS, 100 U/mL penicillin, and 100 mg/mL streptomycin in a humidified incubator of 5% CO_2_ at 37°C.

### 2.9 Transfection of siRNA and Plasmids

Small interfering RNA (siRNA) targeting ChREBP and overexpression plasmids for ChREBP and PGC‐1α were acquired from Guangzhou Hanyi Biotechnology Co. Ltd. (Guangzhou, China). The siRNA interference sequence is provided in Table S1. According to the product instructions, siRNA was delivered into HAPI cells using Namipo transfection reagent (P2307149; Transheep Bio), and plasmids were transfected using Lipofectamine 3000 transfection reagent (L3000‐015; Invitrogen). The transfected HAPI cells were stimulated with 100 ng/mL LPS for 24 h [30].

### 2.10 Flow Cytometry Analysis

HAPI cells were trypsinized, collected, centrifuged, and resuspended. Following the manufacturer's instructions, antibodies including CD86/APC (200315; Biolegend), CD206/FITC (bs‐4727R‐FITC, Bioss), and CD11b/PE (201807; Biolegend) were added to the cells. After being incubated on ice in the dark for 15–20 min, the cells were then centrifuged, resuspended, filtered, and analyzed with a flow cytometer (FACSVerse, BD).

### 2.11 Seahorse XF Cell Energy Metabolism Assay

The oxygen consumption rate (OCR) of microglia was measured using the Seahorse XFe96 Extracellular Flux Analyzer (Agilent, USA). The day prior to the formal experiment, the sensor probe plate was hydrated with Seahorse XF calibrant solution in a non‐CO₂ incubator at 37°C overnight. Meanwhile, HAPI cells were seeded at 10,000 cells per well in the Seahorse XF cell culture microplate. On the day of the experiment, Seahorse XF medium supplemented with 10 mM glucose, 1 mM sodium pyruvate, and 2 mM glutamine was adjusted to pH 7.4 before being added to HAPI cells. Prior to OCR measurement, cells were pretreated with or without 4 μM Etomoxir for 2 h; cellular OCR was then measured under basal conditions and after successive additions of oligomycin (1.5 μM), FCCP (1 μM), and rotenone/antimycin A (0.5 μM) [31]. Afterward, protein content in each well was measured using the BCA protein assay kit (ZJ102; Yaenzyme) to normalize OCR data by cell number.

### 2.12 RNA Extraction and RT‐qPCR

Total RNA was extracted using AG RNAex Pro Reagent (AG21102; AG). mRNA was reverse‐transcribed with Evo M‐MLV Reverse Transcription Premix Kit (AG11728; AG). Gene expression was quantified by SYBR Green Pro Taq HS Premix qPCR Kit (AG11718; AG) and analyzed using the 2^−ΔΔCt^ method with β‐actin as the reference gene. Primer sequences are listed in Table S2.

### 2.13 Western Blot

Proteins were extracted using the total protein extraction kit (KGP250; Kaiji) and quantified with the BCA protein assay kit (ZJ102; Yaenzyme). They were separated by SDS‐PAGE and transferred to PVDF membranes. The membrane was blocked and incubated with primary antibodies, including ChREBP (1:1000, NB400‐135; Novus Biologicals), PGC‐1α (1:5000, 66369‐1‐Ig; Proteintech), CPT1A (1:1000, 15184‐1‐AP; Proteintech), ACADM (1:1000, 55210‐1‐AP; Proteintech), NRF1 (1:1000, 12482‐1‐AP; Proteintech), TFAM (1:1000, 82745‐1‐RR; Proteintech), and β‐actin (1:15000, 66009‐1‐Ig; Proteintech). After incubation with primary antibodies, membranes were incubated with HRP‐conjugated secondary antibodies, including goat anti‐rabbit IgG/HRP (1:5000, SE134; Solarbio) and goat anti‐mouse IgG/HRP (1:5000, SE131; Solarbio). After incubation with secondary antibodies and visualization using ECL reagent (WBKLS0100; Millipore), bands were analyzed with Image J software.

### 2.14 PPI Network Construction and Bioinformatics Analysis

The protein–protein interaction (PPI) network was constructed using the STRING database (https://cn.string‐db.org/) with a minimum interaction score threshold of 0.4. The PPI network was imported into Cytoscape software (version 3.7.1) for hub gene identification and ranking using the degree algorithm. Additionally, the DAVID online tool (https://davidbioinformatics.nih.gov/) was used for functional analyses of these hub genes, focusing on Gene Ontology (GO) classification and Kyoto Encyclopedia of Genes and Genomes (KEGG) pathways.

### 2.15 Dual Luciferase Reporter Gene Assay

Binding sites of ChREBP on PGC‐1α promoter were predicted using the JASPAR website (https://jaspar.elixir.no/). The empty vector (EV) and ChREBP overexpression plasmid were co‐transfected into HAPI cells with either the wild‐type PGC‐1α (WT) or mutated PGC‐1α (MUT1: site 1 mutation; MUT2: site 2 mutation; MUT3: sites 1 + 2 mutation) luciferase reporter plasmids. Measure the luciferase activity of the PGC‐1α using the dual‐luciferase reporter gene assay kit (11402ES60; Yeasen). Firefly luciferase to Renilla luciferase ratio was used to compute relative luciferase activity.

### 2.16 Chromatin Immunoprecipitation (ChIP)

Using the Simple ChIP Enzymatic Chromatin IP Kit (9003S; CST), chromatin immunoprecipitation (ChIP) was performed. Glycine was added after cells were cross‐linked using a 1% formaldehyde solution. Following two washes with PBS, the cells were collected, centrifuged, and resuspended in a solution that included DTT, PMSF, and protease inhibitors. The mixture was treated with micrococcus nuclease, and the reaction was stopped with EDTA. Following centrifugation, the mixture was resuspended in ChIP buffer and subjected to ultrasonication. The sonicated chromatin was incubated with IgG (2729S; CST), histone H3 (4620S; CST), and ChREBP (NB400‐135; Novus Biologicals). ChIP‐grade protein G magnetic beads were added, followed by washing, eluting, and purifying the DNA. RT‐qPCR analysis was performed using the following primers: The forward primer sequence for PGC‐1α was 5′‐CCTTTGGAATGGTTGAGAAGGC‐3′ and the reverse primer sequence was 5′‐TACGTCTCGATTTTCTTCCTGTCCG‐3′. Using the comparative Ct method, the target fragment's fold enrichment was calculated and normalized to the input sample.

### 2.17 Statistical Analysis

For statistical analysis, an unpaired two‐tailed Student's *t*‐test was used to compare data between two groups. When analyzing three or more groups, a one‐way analysis of variance (ANOVA) was conducted with Tukey's post hoc test. In behavior experiments, non‐parametric tests were used to evaluate paw withdrawal thresholds: the Mann–Whitney *U*‐test for two groups' comparisons and the Kruskal–Wallis test for three or more groups [32]. Statistical significance was set at *p* < 0.05. All analyses were performed using SPSS 26.0 (IBM, USA) and GraphPad Prism 9 (GraphPad Software, USA).

## 3 Results

### 3.1 ChREBP Expression Is Upregulated in Microglia of SNI Rats and LPS‐Stimulated HAPI Cells

**FIGURE 1:**
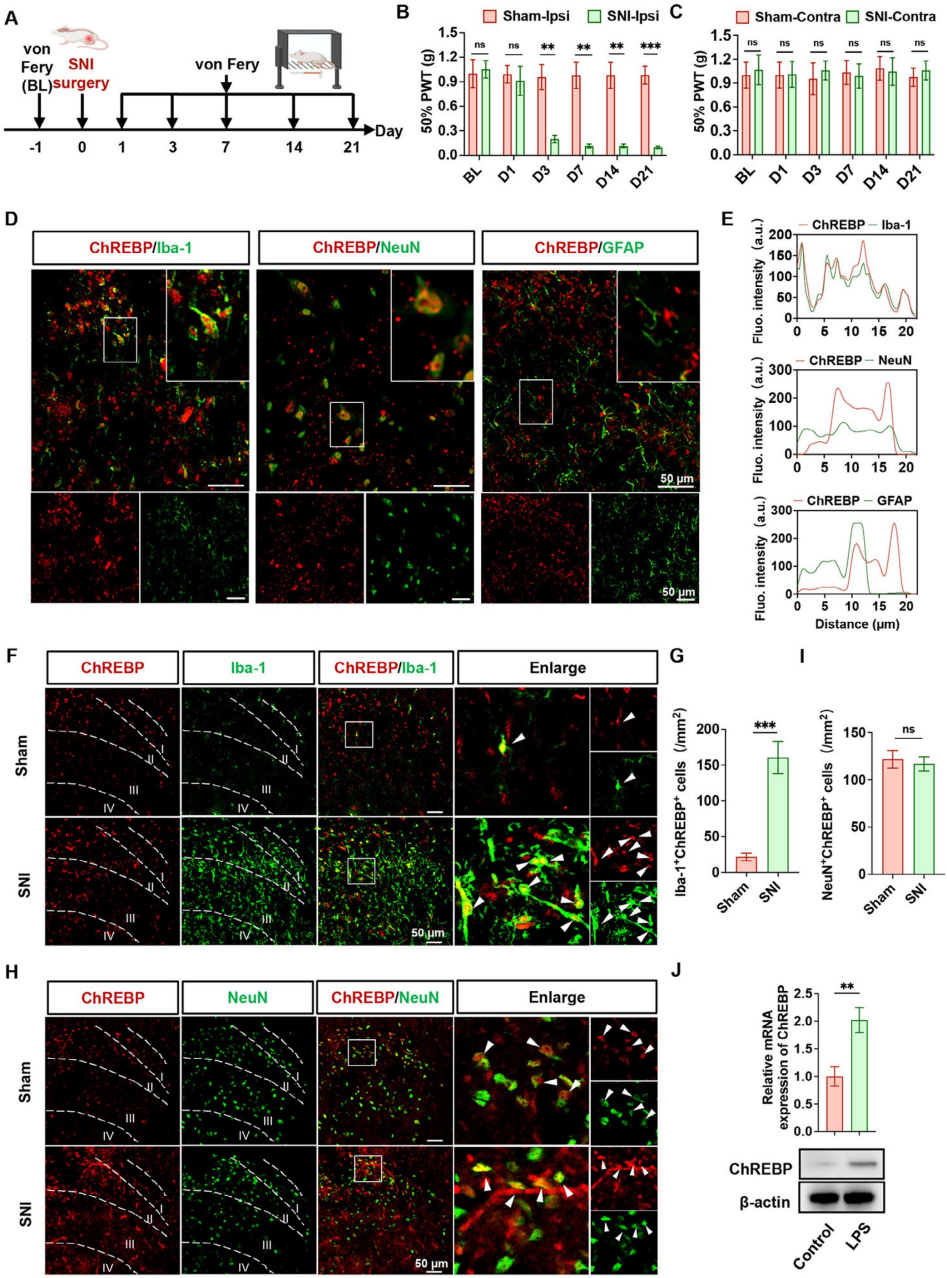
ChREBP expression is increased in microglia of the NP rat model. (A) Timeline of the SNI surgery and behavioral testing. (B, C) Von Frey tests were conducted to measure the mechanical pain threshold in the ipsilateral (B) and contralateral (C) sides of the Sham group and SNI group before SNI surgery (baseline: BL) and on days 1, 3, 7, 14, and 21 after SNI surgery. (D, E) Representative immunofluorescence staining images (D) and colocalization analysis (E) of ChREBP with Iba‐1, ChREBP with NeuN, and ChREBP with GFAP. (F, G) Representative immunofluorescence staining images and quantitative analysis of the number of Iba‐1^+^ChREBP^+^ double‐positive cells in the Sham group and SNI group. (H, I) Representative immunofluorescence staining images and quantitative analysis of the number of NeuN^+^ChREBP^+^ double‐positive cells in the Sham group and SNI group. (J) Protein band image of ChREBP in HAPI cells. Data are presented as mean ± SD, *n* = 6, ^ns^ *p* > 0.05, **p < 0.01, ***p < 0.001.

The NP rat model was established using the SNI method (Figure 1A). After the third day following SNI surgery, the pain threshold on the ipsilateral (Ipsi) side of the rats was significantly decreased and remained low until the 21st day after surgery (Figure 1B). In contrast, the pain threshold on the contralateral (Contra) side did not exhibit significant changes (Figure 1C). We then examined ChREBP expression in the spinal cord following SNI surgery using immunofluorescence and RT‐qPCR. As shown in Figure S1A, the ipsilateral SDH was delineated into laminae I–VI. We found that ChREBP expression was significantly increased in the superficial dorsal horn, with the changes predominantly localized to laminae I–III in SNI rats compared with the sham group (Figure S1A–F). Consistently, ChREBP mRNA levels were also elevated (Figure S1G). Next, we utilized immunofluorescent staining to co‐label ChREBP with the microglial marker Iba‐1, the neuronal marker NeuN, or the astrocytic marker GFAP in SNI rats. We found that ChREBP was predominantly co‐localized with Iba‐1, to a lesser extent with NeuN, and did not co‐localize with GFAP (Figure 1D,E). Additionally, we observed an increased number of Iba‐1^+^ChREBP^+^ cells (Figure 1F,G), while the number of NeuN^+^ChREBP^+^ cells (Figure 1H,I) remained unchanged in the SDH of SNI rats. Therefore, we focused on microglia as key players in the pathology of NP and employed HAPI cells (a microglial cell line). Following LPS stimulation, we observed elevated mRNA levels of tumor necrosis factor‐α (TNF‐α), interleukin‐1β (IL‐1β), and interleukin‐6 (IL‐6) in HAPI cells (Fig. S2A). Additionally, LPS stimulation significantly upregulated ChREBP mRNA levels in HAPI cells (Figure S2B), and western blotting confirmed elevated ChREBP protein levels (Figure 1J). In conclusion, these results indicate that ChREBP is upregulated in the SDH microglia of SNI rats and in HAPI cells exposed to LPS, suggesting a close relationship between ChREBP and NP in microglia.

### 3.2 ChREBP Mitigates Pain by Mediating Microglial Anti‐Inflammatory Polarization and Suppressing Inflammation

To elucidate the role of ChREBP in microglia, we designed siRNA specifically targeting ChREBP and transfected it into HAPI cells exposed to LPS. Post‐transfection, both ChREBP mRNA and protein expression were significantly downregulated (Figure S3A–C). Flow cytometry analysis revealed that ChREBP knockdown led to an increased ratio of CD11b^+^CD86^+^/CD11b^+^CD206^+^ cells, indicating a shift toward a pro‐inflammatory phenotype (Figure S3D,E). Moreover, following ChREBP knockdown, the mRNA levels of TNF‐α, IL‐1β, and IL‐6 were significantly upregulated in LPS‐stimulated HAPI cells (Figure S3F). Conversely, we transfected a ChREBP overexpression plasmid into HAPI cells exposed to LPS. This resulted in a substantial increase in ChREBP mRNA levels, exceeding 30‐fold (Figure S3G), and a significant upregulation of ChREBP protein expression (Figure S3H,I). Flow cytometry analysis demonstrated that ChREBP overexpression decreased the ratio of CD11b^+^CD86^+^/CD11b^+^CD206^+^ cells (Figure S3J,K), suggesting a shift toward an anti‐inflammatory phenotype. Additionally, ChREBP overexpression downregulated the mRNA levels of TNF‐α, IL‐1β, and IL‐6 in LPS‐exposed HAPI cells (Figure S3L). These findings suggest that ChREBP plays a pivotal role in modulating the anti‐inflammatory phenotype of microglia, with potential implications for neuroinflammation.

**FIGURE 2:**
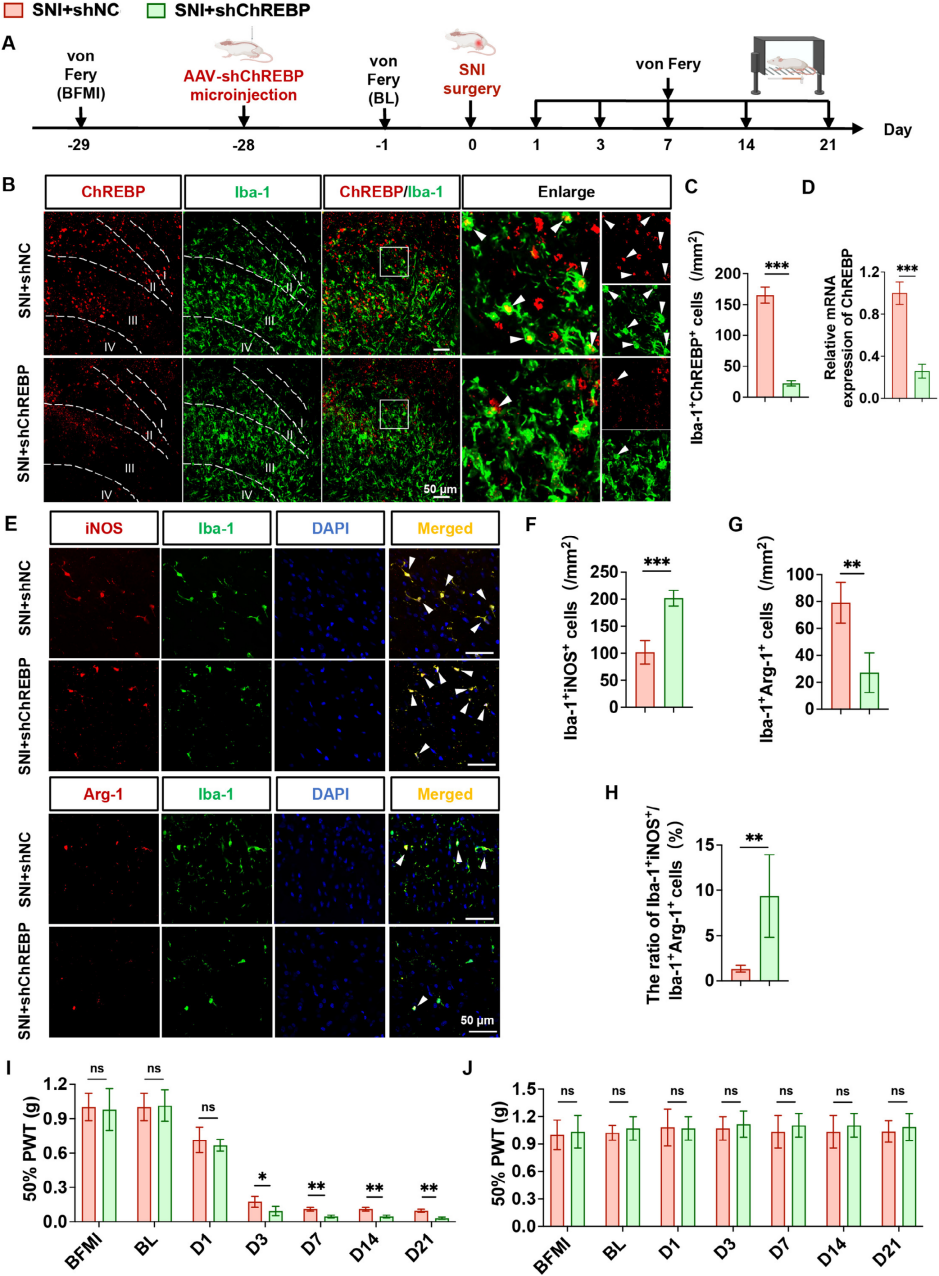
In vivo knockdown of ChREBP reduces the number of anti‐inflammatory microglia and aggravates pain. (A) Experimental timeline of SNI surgery, spinal cord stereotaxic injection, and behavioral tests. (B, C) Representative immunofluorescence staining images (B) and quantitative analysis of cell numbers (C) of ChREBP and Iba‐1‐positive cells in the spinal cord. (D) ChREBP mRNA level in the spinal cord. (E) Representative immunofluorescence staining images for double‐labeled Iba‐1 and pro‐inflammatory microglial marker (iNOS) and double‐labeled Iba‐1 and anti‐inflammatory microglial marker (Arg‐1) in the spinal cord. (F–H) Quantitative analysis of immunofluorescence results, including (F) number of Iba‐1^+^iNOS^+^ colocalized cells; (G) number of Iba‐1^+^Arg‐1^+^ colocalized cells; (H) ratio of Iba‐1^+^iNOS^+^ to Iba‐1^+^Arg‐1^+^ cells. (I–J) Mechanical pain thresholds on the ipsilateral (I) and contralateral (J) sides were measured before microinjection (BFMI), at baseline (BL), and on days 1, 3, 7, 14, and 21 after SNI surgery in rats. Data are presented as mean ± SD, *n* = 6, ^ns^ *p* > 0.05, *p < 0.05, **p < 0.01, ***p < 0.001.

To further confirm the in vivo role of microglial ChREBP in NP, AAV9 vectors containing the F4/80 promoter (AAV9‐F4/80‐shChREBP for ChREBP knockdown and AAV9‐F4/80‐oeChREBP for ChREBP overexpression) were stereotactically injected into the SDH of the ipsilateral side in SD rats, respectively, 4 weeks prior to SNI surgery (Figure 2A and Figure S4A). Immunofluorescence co‐localization experiments (with microglial marker Iba‐1) demonstrated that, in the SNI rat model, injection of AAV‐shChREBP into the ipsilateral SDH significantly decreased ChREBP expression specifically in microglia (Figure 2B,C), while AAV‐ChREBP injection markedly increased microglial ChREBP expression (Figure S4B,C). RT‐qPCR analysis further verified that ChREBP mRNA levels were significantly reduced in SNI rats receiving AAV‐shChREBP and significantly elevated in those receiving AAV‐ChREBP, compared to respective control groups (Figure 2D and Figure S4D). To assess the effect of microglial ChREBP modulation on microglial polarization in vivo, immunofluorescence staining was performed using pro‐inflammatory marker iNOS and anti‐inflammatory marker Arg‐1 (both co‐labeled with Iba‐1). In the SNI + AAV‐shChREBP group, the ratio of Iba‐1^+^iNOS^+^/Iba‐1^+^Arg‐1^+^ cells was significantly increased, indicating a shift toward a pro‐inflammatory microglial phenotype (Figure 2E–H). In contrast, the SNI + AAV‐ChREBP group showed a significant decrease in the Iba‐1^+^iNOS^+^/Iba‐1^+^Arg‐1^+^ ratio, reflecting a shift toward an anti‐inflammatory phenotype (Figure S4E–H). Behavioral tests were conducted to evaluate mechanical pain threshold using von Frey filaments. Results showed that microglial ChREBP knockdown (via AAV‐shChREBP) resulted in a significantly lower mechanical pain threshold on the ipsilateral side at 3, 7, 14, and 21 days post‐SNI, exacerbating NP symptoms (Figure 2I). Conversely, microglial ChREBP overexpression (via AAV‐ChREBP) significantly increased the mechanical pain threshold on the ipsilateral side at 3, 7, 14, and 21 days post‐SNI, alleviating NP (Figure S4I). Importantly, no significant changes in mechanical pain threshold were observed on the contralateral side in AAV‐injected group (Figure 2J and Figure S4J).

Collectively, these in vitro and in vivo findings highlight the regulatory role of ChREBP in microglial phenotype: it promotes anti‐inflammatory polarization and suppresses pro‐inflammatory responses, thereby alleviating SNI‐induced NP.

### 3.3 ChREBP Enhances Fatty Acid Oxidation Rate and Energy Production in Microglia

**FIGURE 3:**
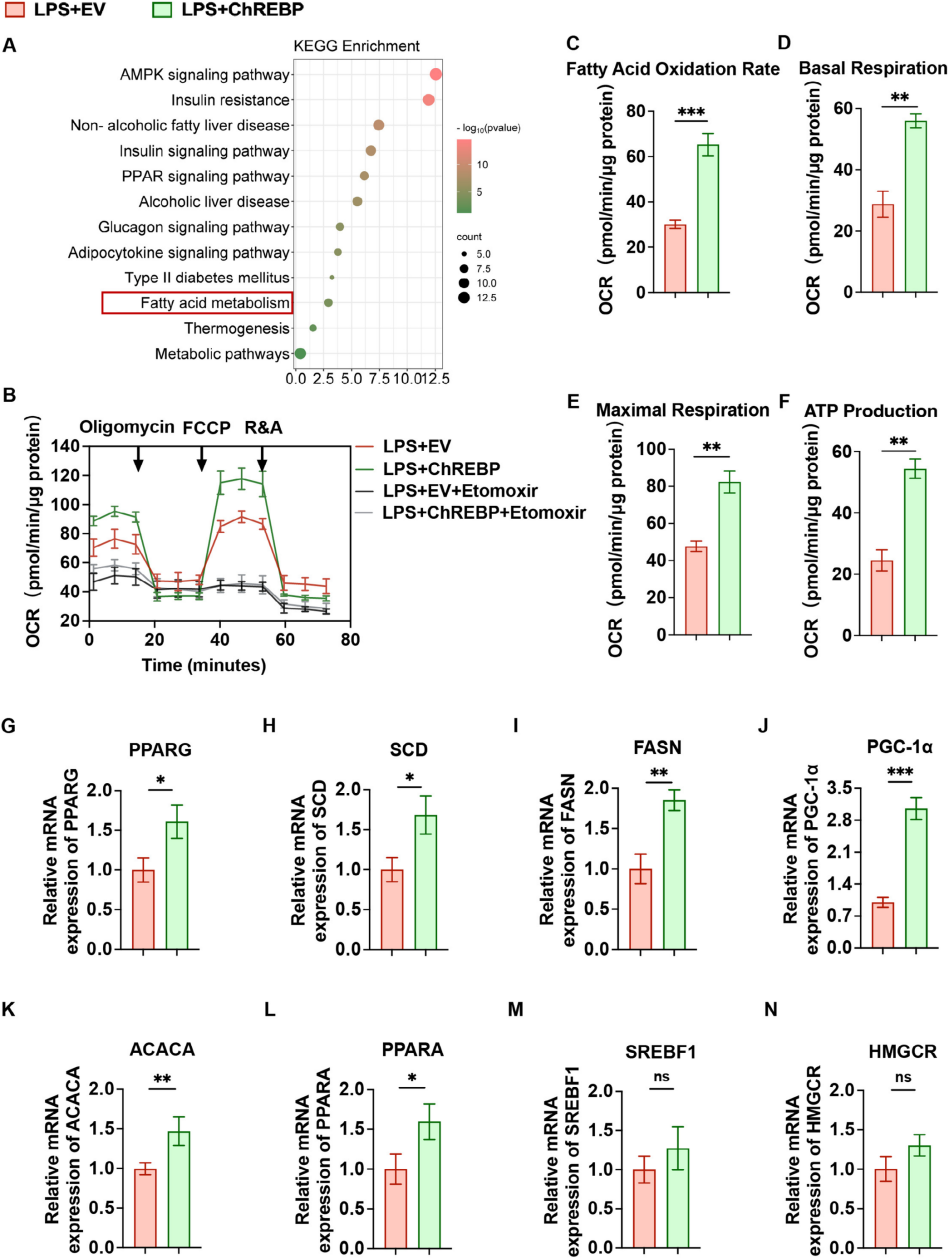
Overexpression of ChREBP activates the fatty acid oxidation in microglia. (A) KEGG enrichment analysis of the 50 hub genes significantly associated with ChREBP. (B) This panel compares the oxygen consumption rate of HAPI cells in four groups: Empty vector plasmid (±Etomoxir) and ChREBP overexpression plasmid (±Etomoxir). (C–F) Effects of ChREBP overexpression on fatty acid oxidation rate (C), basal respiration (D), maximal respiration (E), and ATP production (F) in the HAPI cells. (G‐N) RT‐qPCR was used to detect the mRNA expression levels of PPARG (G), SCD (H), FASN (I), PGC‐1α (J), ACACA (K), PPARA (L), SREBF1 (M), and HMGCR (N) in HAPI cells. These eight hub genes were selected from the STRING‐derived candidate gene set based on their highest connectivity degrees in the Cytoscape PPI network. Data are presented as mean ± SD, *n* = 3, ^ns^ *p* > 0.05, *p < 0.05, **p < 0.01, ***p < 0.001.

To elucidate the mechanisms by which ChREBP regulates microglial polarization and function, we identified the top 50 ChREBP‐associated genes from the STRING database (Figure S5A). Subsequently, we performed GO functional annotation and KEGG enrichment analysis of these ChREBP‐related genes. GO functional annotation results showed that: in terms of biological processes, the ChREBP‐related genes were significantly enriched in terms related to fatty acid homeostasis, cellular response to insulin stimulus, and glucose homeostasis; for cellular components, the enriched terms included chromatin, the SREBP‐SCAP‐Insig complex, and the cytosol; and for molecular functions, the key functional term was RNA polymerase II sequence‐specific DNA‐binding transcription factor binding (Figure S6A). KEGG enrichment analysis further indicated that these ChREBP‐related genes are primarily associated with the AMPK signaling pathway, insulin resistance, fatty acid metabolism, and metabolic pathways (Figure 3A). Given that these pathways are all closely linked to fatty acid metabolism, these results indicate that ChREBP is primarily involved in this metabolic pathway in microglia. To evaluate the impact of ChREBP on microglial fatty acid metabolism, we employed a Seahorse XF extracellular flux analyzer to measure the OCR, a key indicator of mitochondrial respiration and energy metabolism. Results demonstrated that ChREBP overexpression significantly promoted the fatty acid oxidation rate, as well as basal respiration, maximal respiration, and ATP production in LPS‐exposed HAPI cells (Figure 3B–F). Collectively, these findings demonstrate that ChREBP plays a crucial role in microglial energy metabolism.

Hub genes were selected from candidate genes retrieved from the STRING database through a filtering process based on connectivity degrees calculated using Cytoscape software. The detailed connectivity values for each gene are provided in Table S3. RT‐qPCR experiments were conducted to validate the top eight hub genes ranked by connectivity degree in the Cytoscape‐based PPI network, which are shown in Figure 3G–N. The results showed that ChREBP significantly increased the mRNA levels of PPARG (Figure 3G), SCD (Figure 3H), FASN (Figure 3I), PGC‐1α (Figure 3J), ACACA (Figure 3K), and PPARA (Figure 3L). SREBF1 (Figure 3M) and HMGCR (Figure 3N) exhibited a slight upregulation trend, although this was not statistically significant. Notably, PGC‐1α displayed the most pronounced increase in expression. Consistent with its role as a master regulator of fatty acid oxidation, this upregulation likely contributes to the higher fatty acid oxidation rate observed in ChREBP‐overexpressing cells, further supporting the link between ChREBP and microglial fatty acid metabolism.

### 3.4 ChREBP Directly Regulates PGC‐1α Expression by Binding to Its Promoter Region

**FIGURE 4:**
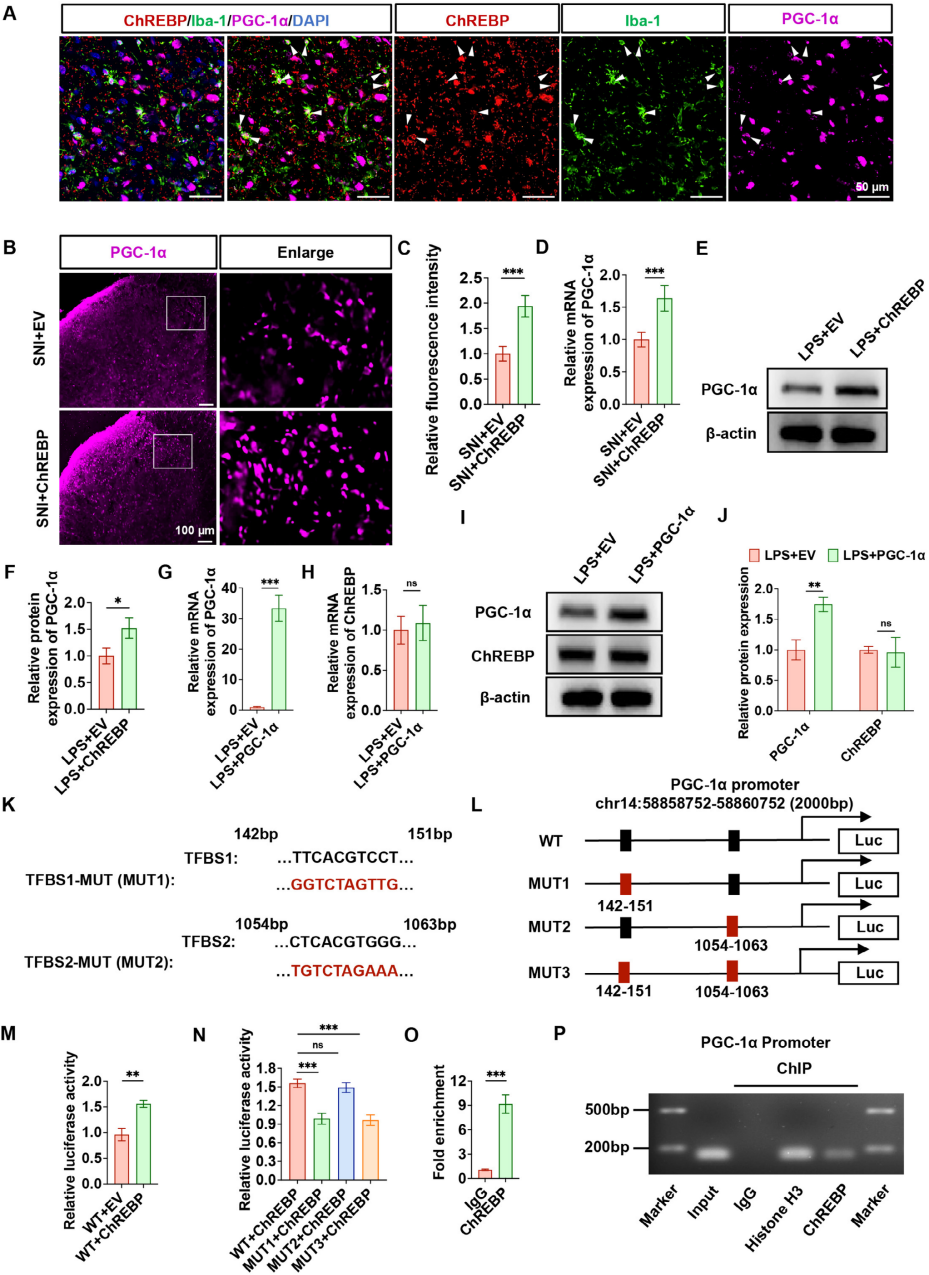
ChREBP directly regulates PGC‐1α expression by binding to its promoter region. (A) Both ChREBP and PGC‐1α colocalize with Iba‐1 in the spinal cord of NP rats. (B‐C) Representative immunofluorescence images of PGC‐1α after in vivo overexpression of ChREBP (B) and quantitative analysis of fluorescence intensity (C). (D) mRNA level of PGC‐1α after in vivo overexpression of ChREBP. (E, F) Effect of ChREBP overexpression on PGC‐1α protein level in HAPI cells (E) and quantitative analysis of protein bands (F). (G, H) mRNA levels of PGC‐1α (G) and ChREBP (H) after overexpression of PGC‐1α in HAPI cells. (I, J) Protein expression of PGC‐1α and ChREBP after overexpression of PGC‐1α in HAPI cells (I), and quantitative analysis of protein bands (J). (K) Two binding sites between ChREBP and the PGC‐1α promoter region were predicted using the JASPAR database. (L) Schematic diagram of the constructed luciferase reporter plasmids for the wild‐type (WT) and mutant (MUT1, MUT2, MUT3) PGC‐1α promoters. (M) Dual‐luciferase reporter assay showing the effect of ChREBP overexpression on luciferase activity of the WT PGC‐1α promoter plasmid. (N) Dual‐luciferase reporter assay comparing the effect of ChREBP overexpression on luciferase activity between the WT and three mutant (MUT1, MUT2, MUT3) PGC‐1α promoter plasmids. (O) ChIP‐qPCR showing the amplification of PGC‐1α promoter fragments in the ChREBP group compared with the IgG group. (P) The enrichment of PGC‐1α promoter fragments by ChREBP immunoprecipitation. Marker: DNA marker; Input: 2% input sample; IgG: Negative control; Histone H3: Positive control. ChREBP: Specific antibody for target detection. Data are represented as mean ± SD, *n* = 6 for in vivo experiments (A–D), *n* = 3 for in vitro experiments (E‐P), ^ns^ *p* > 0.05, *p < 0.05, **p < 0.01, ***p < 0.001.

Immunofluorescence analysis showed that ChREBP and PGC‐1α co‐localized with Iba‐1 in the SDH of the SNI rat model (Figure 4A). Furthermore, in vivo upregulation of ChREBP promoted increased PGC‐1α expression within the ipsilateral SDH of SNI rats with ChREBP overexpression (Figure 4B,C). Moreover, PGC‐1α mRNA levels were upregulated in SNI rats with ChREBP overexpression (Figure 4D). We observed that ChREBP overexpression led to a significant elevation in the protein level of PGC‐1α in HAPI cells (Figure 4E,F). However, when the PGC‐1α overexpression plasmid was transfected into HAPI cells exposed to LPS, PGC‐1α overexpression was found to have little effect on ChREBP mRNA and protein levels (Figure 4G–J). According to these results, we think that ChREBP most likely acts upstream of PGC‐1α.

To further confirm whether this regulatory effect is direct, we performed a series of experiments. The JASPAR database was used to identify transcription factor binding sites (TFBS) for ChREBP in the PGC‐1α promoter region. TFBS 1 is located between base pairs 142 and 151, while TFBS 2 is situated between base pairs 1054 and 1063 (Figure 4K). We then constructed PGC‐1α mutant plasmids, with PGC‐1α‐MUT 1 targeting TFBS 1, PGC‐1α‐MUT 2 targeting TFBS 2, and PGC‐1α‐MUT 3 containing mutations in both TFBS 1 and TFBS 2 (Figure 4L). Dual‐luciferase reporter gene assay revealed that ChREBP significantly enhanced the luciferase activity of PGC‐1α (Figure 4M), and TFBS 1 was identified as the primary binding site for ChREBP on the PGC‐1α promoter (Figure 4N). ChIP assay was performed using PGC‐1α promoter primers to detect ChIP DNA products. The results revealed that ChREBP increased the amplification of PGC‐1α promoter fragments (Figure 4O,P). These findings suggest that ChREBP directly regulates PGC‐1α expression through binding to its promoter region.

### 3.5 ChREBP Facilitates Fatty Acid Oxidation and Anti‐Inflammatory Phenotype in SNI Rats via PGC‐1α

**FIGURE 5:**
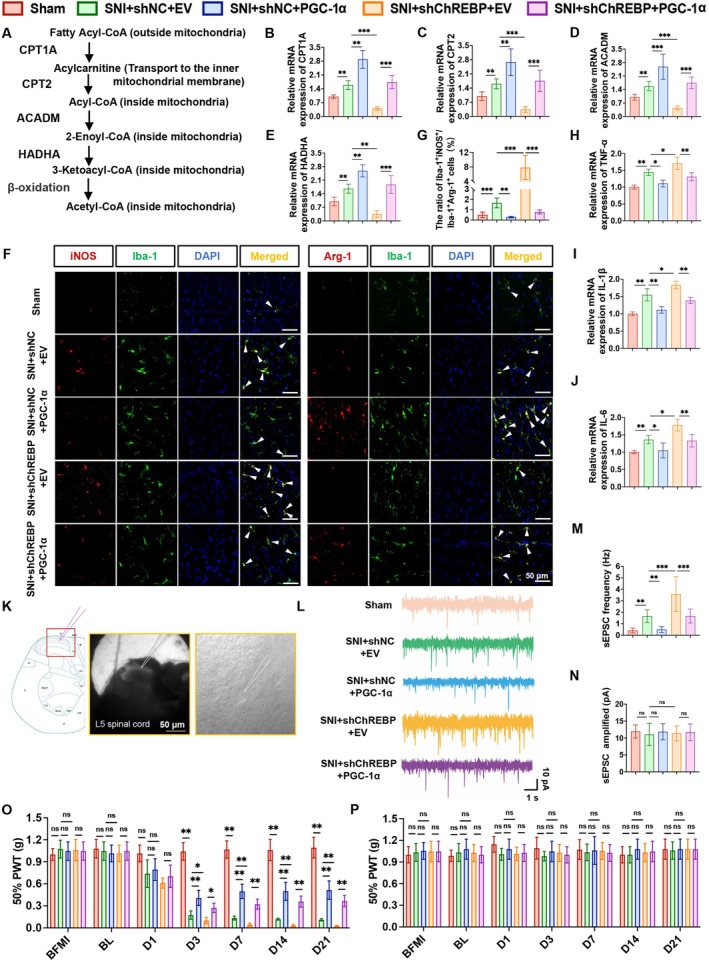
PGC‐1α overexpression reverses the microglial metabolism–polarization–inflammation–excitability–pain axis induced by ChREBP knockdown in microglia. (A) Schematic diagram showing the role of key molecules in the fatty acid oxidation pathway. (B‐E) mRNA levels of key fatty acid oxidation molecules CPT1A (B), CPT2 (C), ACADM (D), and HADHA (E) in the spinal cord of rats in each group. (F, G) Representative immunofluorescence staining images for double‐labeled Iba‐1 and pro‐inflammatory microglial marker (iNOS), and double‐labeled Iba‐1 and anti‐inflammatory microglial marker (Arg‐1) in the spinal cord (F), and the ratio of Iba‐1^+^iNOS^+^ to Iba‐1^+^Arg‐1^+^ cells (G). (H–J) mRNA levels of inflammatory factors TNF‐α (H), IL‐1β (I), and IL‐6 (J) in the spinal cord of rats in each group. (K) Patch‐clamp electrophysiological recordings performed at the L5 spinal cord segment. (L–N) Representative images of spontaneous excitatory postsynaptic currents (sEPSC) in the spinal cord of rats in each group (L), and statistical analysis of their frequency (M) and amplitude (N). (O, P) Mechanical pain thresholds on the ipsilateral (O) and contralateral (P) sides of rats in each group. Data are represented as mean ± SD; *n* = 6 for in vivo experiments (B–J, O, P), *n* = 3 for electrophysiological recordings (K–N), ^ns^ *p* > 0.05, *p < 0.05, **p < 0.01, ***p < 0.001.

During the fatty acid oxidation process, long‐chain acyl‐CoA outside mitochondria is catalyzed by CPT1A to form acylcarnitine. After acylcarnitine is transported across the inner mitochondrial membrane, it is re‐catalyzed by CPT2 to generate acyl‐CoA. The acyl‐CoA entering mitochondria sequentially undergoes reactions involving enzymes such as ACADM and HADHA and finally produces acetyl‐CoA through β‐oxidation (Figure 5A). RT‐qPCR was performed to detect the mRNA levels of key fatty acid oxidation molecules (CPT1A, CPT2, ACADM, and HADHA) in the spinal cord of rats from each group (Figure 5B–E). The results showed that the downregulation of these key fatty acid oxidation molecules induced by ChREBP knockdown could be partially reversed by PGC‐1α overexpression. To further corroborate that the ChREBP–PGC‐1α axis regulates fatty acid oxidation and mitochondrial biogenesis at the protein level, we assessed CPT1A, ACADM, and the mitochondrial biogenesis regulators NRF1 and TFAM in LPS‐exposed HAPI microglia. ChREBP knockdown decreased CPT1A, ACADM, NRF1, and TFAM protein levels, whereas additional PGC‐1α overexpression significantly increased the protein levels of these molecules (Figure S7A–G), supporting that ChREBP promotes microglial fatty acid oxidation and mitochondrial biogenesis via PGC‐1α. Immunofluorescence staining results revealed that, in the ChREBP knockdown group, the ratio of iNOS^+^Iba‐1^+^/Arg‐1^+^Iba‐1^+^ cells was increased. This phenotypic change could also be partially restored by PGC‐1α overexpression (Figure 5F,G). Additionally, the upregulation of pro‐inflammatory cytokine (TNF‐α, IL‐1β, and IL‐6) mRNA expression triggered by ChREBP knockdown was similarly partially reversed via PGC‐1α overexpression (Figure 5H–J). For the detection of neuronal excitability, sEPSCs of neurons in lamina II of the SDH at the L5 segment were recorded (Figure 5K). It was found that the increase in sEPSC frequency caused by ChREBP knockdown could be partially reversed by PGC‐1α overexpression, while there was no significant difference in sEPSC amplitude among the groups (Figure 5L–N). Behavioral testing results indicated that the reduction in 50% PWT on the ipsilateral side after SNI modeling (e.g., on and after post‐operative day 3) induced by ChREBP knockdown could be partially reversed by PGC‐1α overexpression (Figure 5O). In contrast, there was no significant difference in 50% PWT on the contralateral side across all groups (Figure 5P). In conclusion, ChREBP alleviates pain symptoms in SNI rat models through PGC‐1α by promoting fatty acid oxidation, enhancing anti‐inflammatory microglial polarization, ameliorating inflammatory injury, and reducing the excitability of spinal neurons. These findings indicate that PGC‐1α upregulation is an important downstream mechanism through which ChREBP exerts its protective effects in NP.

### 3.6 ChREBP Inhibits Inflammatory Injury, Suppresses Spinal Neuronal Excitability, and Alleviates Pain in SNI Rats by Regulating Fatty Acid Oxidation

**FIGURE 6:**
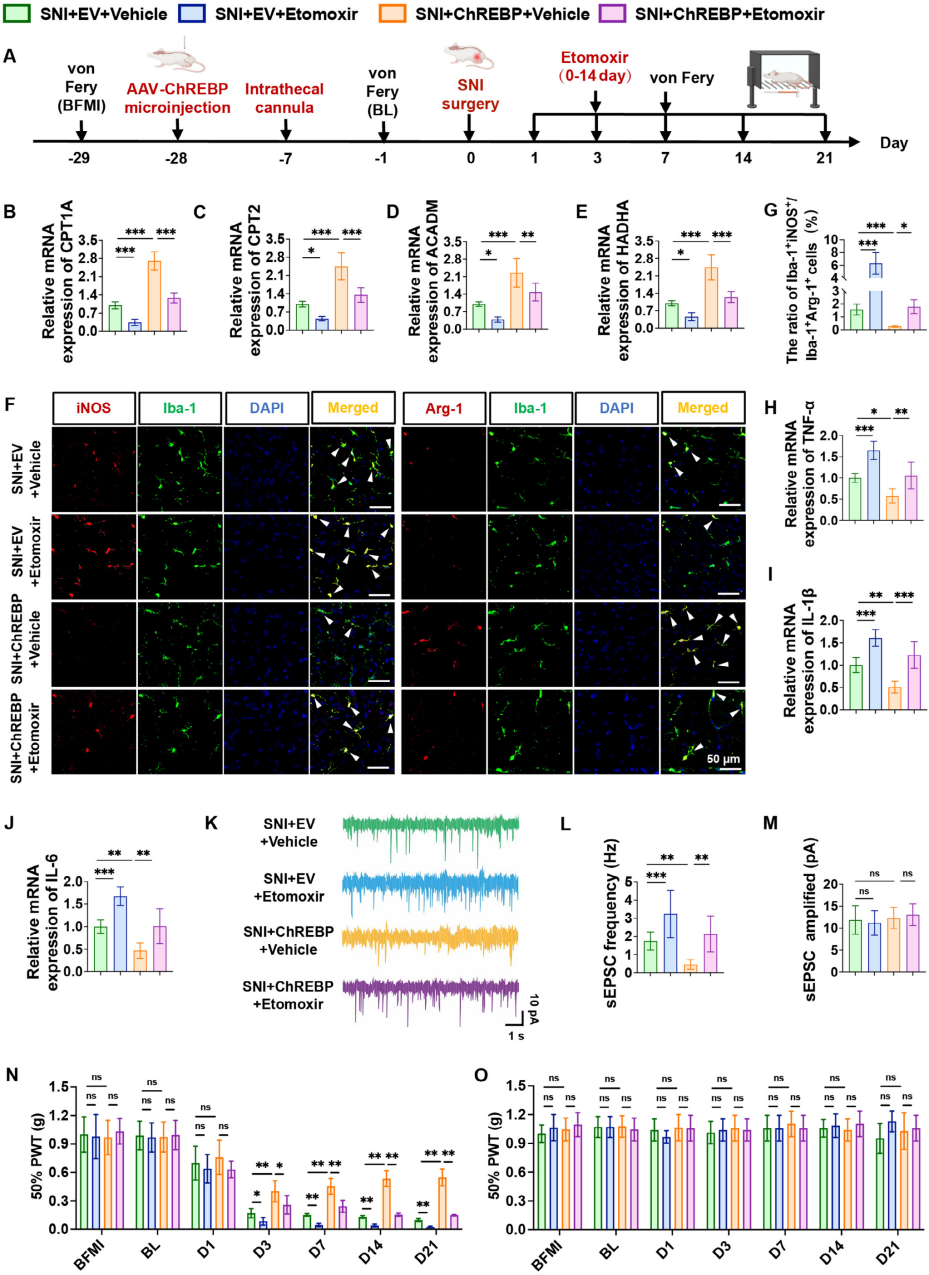
Etomoxir eliminates the protective effect of ChREBP overexpression in microglia on the microglial metabolism–polarization–inflammation–excitability–pain axis by inhibiting microglial fatty acid oxidation. (A) Experimental timeline of SNI surgery, spinal cord stereotaxic injection, intrathecal catheterization, and behavioral tests. (B–E) mRNA levels of key fatty acid oxidation molecules CPT1A (B), CPT2 (C), ACADM (D), and HADHA (E) in the spinal cord of rats in each group. (F, G) Representative immunofluorescence staining images for double‐labeled Iba‐1 and pro‐inflammatory microglial marker (iNOS), and double‐labeled Iba‐1 and anti‐inflammatory microglial marker (Arg‐1) in the spinal cord (F), and the ratio of Iba‐1^+^iNOS^+^ to Iba‐1^+^Arg‐1^+^ cells (G). (H–J) mRNA levels of inflammatory factors TNF‐α (H), IL‐1β (I), and IL‐6 (J) in the spinal cord of rats in each group. (K–M) Representative images of sEPSC in the spinal cord of rats in each group (K), and statistical analysis of their frequency (L) and amplitude (M). (N, O) Mechanical pain thresholds on the ipsilateral (N) and contralateral (O) sides of rats in each group. Data are represented as mean ± SD; *n* = 6 for in vivo experiments (B–J, N, O), *n* = 3 for electrophysiological recordings (K–M), ^ns^ *p* > 0.05, *p < 0.05, **p < 0.01, ***p < 0.001.

This part of the study aimed to investigate the effects of ChREBP and the fatty acid oxidation inhibitor Etomoxir on SNI rats, and the experimental procedure is shown in Figure 6A. RT‐qPCR results showed that ChREBP overexpression significantly upregulated the mRNA levels of key fatty acid oxidation molecules CPT1A, CPT2, ACADM, and HADHA in spinal tissue, while Etomoxir treatment obviously attenuated this promoting effect (Figure 6B–E). Immunofluorescence staining revealed that Etomoxir partially reversed the inhibitory effect of ChREBP overexpression on pro‐inflammatory microglia (Figure 6F,G). Furthermore, Etomoxir similarly partially reversed the inhibitory effect of ChREBP overexpression on pro‐inflammatory cytokines (TNF‐α, IL‐1β, and IL‐6) (Figure 6H–J). Electrophysiological recordings demonstrated that Etomoxir counteracted the inhibitory effect of ChREBP overexpression on sEPSC frequency, while there was no significant difference in sEPSC amplitude among groups (Figure 6K–M). Behavioral tests indicated that Etomoxir eliminated the analgesic effect of ChREBP overexpression on the ipsilateral side, with no significant changes on the contralateral side (Figure 6N,O). In summary, ChREBP mitigates pain in SNI rats by enhancing fatty acid oxidation, promoting anti‐inflammatory microglial polarization, and reducing neuronal hyperexcitability, whereas these protective effects are notably attenuated by Etomoxir.

## 4 Discussion

By establishing a rat model of NP, this study reveals the specific mechanism by which ChREBP exerts neuroprotective effects in microglia through regulating fatty acid oxidation. First, in the SNI‐induced NP model, ChREBP expression was upregulated in Iba‐1^+^ microglia within the superficial SDH, with the most prominent changes localized to laminae I–III of the ipsilateral L4–L6 segments. Functional experiments confirmed that overexpression of ChREBP significantly inhibits neuroinflammatory responses and alleviates pain, while specific inhibition of ChREBP reverses this protective effect, leading to aggravated inflammation and deterioration of pain symptoms. Second, mechanistic studies demonstrated that ChREBP can directly bind to the TFBS1 site in the promoter region of PGC‐1α and activate its transcription. Third, this study is the first to systematically clarify the role of fatty acid oxidation in the occurrence and development of NP, providing a new theoretical basis and therapeutic target for the metabolic‐immune regulatory mechanism of NP.

NP is characterized by the development and maintenance of persistent pain hypersensitivity, with neuronal hyperexcitability in the SDH recognized as its pivotal pathological mechanism [33]. Among various electrophysiological techniques used to assess the excitability of SDH neurons, sEPSCs serve as a core indicator for elucidating the pathological mechanisms of NP [34]. Accumulating evidence has demonstrated that the excessive release of proinflammatory cytokines in the SDH following SNI surgery constitutes a key driving factor contributing to the abnormal elevation of sEPSCs [35, 36]. Our findings further confirm that following SNI surgery, as the level of inflammation significantly increases, sEPSC frequency correspondingly rises, which is fully consistent with the expected pathophysiological patterns of change. A study has found that TNF‐α could significantly increase the frequency of sEPSCs in layer II neurons of the SDH [37]. In addition, the imbalance of IL‐1β is also closely related to abnormal synaptic excitability [38]. Although the role of IL‐6 in pain regulation is controversial [39], research has clearly confirmed that IL‐6 could induce hyperalgesia by inhibiting the transmission of inhibitory neurotransmitters [40]. In conclusion, during the occurrence and development of NP, proinflammatory factors in the SDH region may jointly regulate neuronal excitability through different signal pathways, thereby becoming a key link in promoting the pathological process of NP.

Microglia, as resident immune cells of the central nervous system, originate from the primitive myeloid progenitors in the yolk sac and migrate to the central nervous system during early embryonic development [41]. In the early stage of peripheral nerve injury, microglia are rapidly activated and secrete an appropriate amount of inflammatory factors to exert neuroprotective effects [42, 43]. In the middle and late stages of peripheral nerve injury, microglia secrete a large number of inflammatory cytokines, leading to excessive neuroinflammation, which further induces microglial activation and functional impairment [44]. In the SNI model of peripheral nerve injury, pathological features such as nutrient deprivation and local microenvironment alterations are observed [45, 46]. Microglia may upregulate genes involved in fatty acid oxidation and switch to utilizing locally accumulated free fatty acids—such as those derived from degenerating myelin sheaths—as an alternative energy source [47]. This metabolic adaptation is not only a survival strategy of microglia under stressful conditions but also helps maintain their anti‐inflammatory and repair phenotype [48]. ChREBP acts as a metabolic sensor in this process [49]. When detecting elevated local fatty acid levels or changes in energy demand, it initiates a protective regulatory program by upregulating CPT1A and CPT2, thereby maintaining fatty acid oxidation [50]. This mechanism explains why ChREBP could inhibit the transformation of microglia to a pro‐inflammatory phenotype, reduce the release of IL‐1β and TNF‐α, further alleviate the inflammatory response, and delay the progression of NP.

Fatty acid oxidation is necessary for anti‐inflammatory microglia, and enhancing fatty acid oxidation endows microglia with anti‐inflammatory properties [51]. Research by Luo et al. demonstrated that when fatty acid oxidation is inhibited using Etomoxir, the activity of Arg‐1, a marker of alternatively activated anti‐inflammatory microglia, decreases significantly [52]. Similarly, oligomycin and FCCP, which inhibit oxidative phosphorylation and uncouple mitochondrial respiration, respectively, have also been confirmed to significantly reduce Arg‐1 activity [53]. A previous study uncovered that ChREBP participated in the fatty acid β‐oxidation and subsequent ketogenesis [54]. Evidence has been provided that mice with ChREBP gene deletion diminished the production of fatty acid oxidation, ketone formation, and FGF 21 [55]. Our findings demonstrate that the beneficial effects of ChREBP, including an increase in anti‐inflammatory microglia, suppression of neuroinflammation, and alleviation of pain, are partially abolished by Etomoxir. This indicates that ChREBP exerts its protective functions primarily through enhanced fatty acid oxidation.

ChREBP is predominantly expressed in metabolic tissues such as the liver, white and brown adipose tissue, intestine, and pancreatic β cells [56], while its presence in neural cell types has been less explored. Nevertheless, emerging evidence indicates that ChREBP also plays significant roles in the nervous system [16, 57, 58]. Notably, we found that ChREBP is expressed in microglia using the human protein atlas (HPA) databases. Subsequently, our experimental results showed an elevation of ChREBP in microglia within the SDH on the ipsilateral side of SNI rats. Importantly, this upregulation appears to reflect an active protective compensatory mechanism rather than a passive consequence of the disease process. This conclusion is strongly supported by AAV‐mediated functional experiments. Overexpression of ChREBP could mimic the endogenous protective effect, reducing the number of pro‐inflammatory microglia and alleviating mechanical hyperalgesia. In contrast, knockdown of ChREBP blocks this protective effect, leading to an increase in the number of pro‐inflammatory microglia and exacerbation of pain. These findings broaden the current understanding of ChREBP's biological functions. In SNI rats, microglial activation and neuronal excitation lead to a sharp increase in energy demand [59, 60]. The upregulation of ChREBP may represent an adaptive response of the central nervous system to this energy crisis [61]. By promoting specific metabolic pathways, ChREBP may help restore local energy balance and suppress harmful inflammatory responses [62, 63]. These findings provide new experimental evidence for elucidating the non‐classical functions of ChREBP in the nervous system.

Beyond our findings, accumulating evidence indicates that PGC‐1α is involved in both the pathogenesis and resolution of chronic pain conditions. In chronic constriction injury (CCI) models, activation of spinal PGC‐1α has been shown to attenuate ROS‐driven mitochondrial dysfunction, rebalance microglial polarization, suppress NLRP3 inflammasome activation, and thereby alleviate mechanical allodynia [64]. Consistent with this, Sestrin2 overexpression has been reported to attenuate cancer‐induced bone pain by activating the AMPK/PGC‐1α axis, which promotes mitochondrial biogenesis and suppresses neuroinflammation [65]. In aging‐related pain, downregulation of PGC‐1α has been linked to persistent mitochondrial stress, exaggerated neuroinflammation, and the transition from acute to chronic pain [66]. By contrast, pharmacological or genetic activation of PGC‐1α mitigates pain by preserving GABAergic interneuron survival in the SDH [67]. In addition, studies show that epigenetic repression of PGC‐1α (e.g., via Sp1/HDAC2‐dependent mechanisms) or disruption of upstream pathways such as TSPO–AMPK–PGC‐1α aggravates NP, whereas restoration of PGC‐1α signaling reverses mechanical hypersensitivity [68, 69]. Taken together, these reports support the view that PGC‐1α is a nodal regulator, providing an important framework for interpreting the mechanisms identified in our study.

PGC‐1α is a central regulator of fatty acid oxidation, mitochondrial biogenesis, and cellular metabolism [70]. Several studies have shown that PGC‐1α overexpression upregulates CPT1A and ACADM, thereby promoting fatty acid oxidation [71, 72]. Whereas loss of PGC‐1α is associated with reduced expression of metabolic genes and impaired fatty acid oxidation and oxidative phosphorylation [73]. A recent study demonstrates that PGC‐1α expression can restore mitochondrial dysfunction by increasing mtDNA content, stabilizing mitochondrial membrane potential, and reducing mitochondrial ROS production [74]. PGC‐1α also promotes mitochondrial biogenesis and suppresses apoptotic signaling through co‐activation of NRF1 and TFAM [75, 76]. A study has shown that Nrf2‐mediated induction of PGC‐1α in CCI models enhances mtDNA content and restores mitochondrial function in the spinal cord, accompanying relief of mechanical allodynia [77]. Although most early work focused on neuronal PGC‐1α [78, 79], more recent studies indicate that microglial PGC‐1α likewise exerts potent anti‐inflammatory and neuroprotective actions [64, 80]. For example, in a mouse model of ischemic stroke, microglia‐specific PGC‐1α overexpression suppresses NLRP3 inflammasome activation; reduces TNF‐α, IL‐1β, and IL‐6 production; enhances mitophagy; and significantly improves neurological outcomes [80]. Whereas in CCI models, spinal PGC‐1α activation alleviates mechanical allodynia in part by normalizing microglial polarization [64]. Consistent with these experimental data, recent reviews have identified microglial PGC‐1α as a pivotal regulator of mitochondrial quality control, mitophagy, and the amelioration of neuroinflammation [81]. Notably, ChREBP and PGC‐1α are transcriptional coactivators and jointly participate in regulating a series of genes related to cell metabolism [82]. In line with this, our data demonstrate that ChREBP directly increases PGC‐1α transcription by binding to its promoter region and that ChREBP facilitates microglial fatty acid oxidation via PGC‐1α. Collectively, these findings underscore a critical ChREBP‐PGC‐1α regulatory axis that modulates fatty acid oxidation, with multifaceted significance in the regulation of neuroinflammation and the maintenance of neuronal homeostasis.

Certainly, this study also has some limitations. First, we used only male SD rats and did not include female animals, so potential sex differences in the ChREBP regulatory mechanism in NP cannot be ruled out. The choice of male rats was based on two considerations: in the classical SNI model, male rats show more stable mechanical allodynia with smaller inter‐individual variability and restricting the initial mechanistic exploration to a single sex reduces additional confounding factors. Male and female animals appear to rely on distinct immune pathways for pain hypersensitivity: spinal microglia are critical mediators in males, whereas adaptive immune cells, particularly T cells, play a more prominent role in females [83]. Moreover, estrogen has been shown to suppress glial activation and neuroinflammation, alleviating injury‐induced NP in female rodents, a regulatory mechanism largely absent in males [84]. In addition, mechanical allodynia assessed by the von Frey test was used as the primary behavioral measure, and we did not systematically evaluate other important pain phenotypes, such as thermal hyperalgesia or spontaneous pain. Thus, our future studies will incorporate female animals and include a broader battery of behavioral tests to determine whether ChREBP‐dependent mechanisms and microglial metabolic regulation generalize across sexes and across different modalities of NP.

Second, although ChREBP is predominantly upregulated in microglia, it is also detectable in neurons within the SDH. Since we did not perform neuron‐specific gain‐ or loss‐of‐function manipulations of ChREBP, we cannot exclude a potential contribution of neuronal ChREBP to NP. Future studies using neuron‐specific genetic interventions will be required to further elucidate the functional differences of ChREBP across distinct cell types. Regarding our viral manipulation, we also acknowledge that F4/80 is expressed not only by resident microglia in the SDH but also by infiltrating monocyte‐derived macrophages following nerve injury. Consequently, our F4/80‐driven AAV9 manipulation may have targeted a broader population of myeloid cells rather than being strictly microglia‐specific. In future studies, we plan to employ more stringent microglia‐targeting strategies, such as Cre‐loxP–based conditional models, to achieve more precise cell‐type‐specific manipulation and further exclude potential contributions from peripheral immune cells.

Third, the mechanistic study focused on ChREBP's transcriptional regulation of PGC‐1α, without examining the specific binding sites through which PGC‐1α regulates its downstream targets. Significantly, previous work has reported that PGC‐1α interacts with CEBPB to promote CPT1A transcription, indicating that PGC‐1α functions within a transcriptional complex to activate fatty acid oxidation genes [85]. In this study, we did not perform ChIP‐qPCR to confirm direct occupancy of PGC‐1α at the CPT1A promoter, and thus, the precise downstream mechanism remains to be elucidated. Future studies using ChIP‐qPCR, CUT&RUN, or related chromatin assays will be necessary to clarify how PGC‐1α is enriched at fatty acid oxidation‐related gene promoters and to identify the binding sites. Additionally, although Etomoxir was used to inhibit CPT1A and assess the role of fatty acid oxidation in ChREBP‐mediated effects, we acknowledge that it may exert off‐target effects on mitochondrial respiratory chain activity. We did not perform the ChREBP knockdown and CPT1A overexpression rescue experiment, and we recognize this as an important avenue for future work to further validate the specificity of the fatty acid oxidation pathway in mediating ChREBP's protective effects.

Fourth, this study primarily demonstrates that the ChREBP–PGC‐1α axis upregulates genes associated with fatty acid oxidation and mitochondrial biogenesis. However, mitochondrial quality (e.g., mtDNA copy number) and function (e.g., membrane potential) were not directly assessed. In addition, the potential anti‐inflammatory role of ketone body production, such as β‐hydroxybutyrate (BHB), was not evaluated. Emerging evidence indicates that microglia under pathological conditions can accumulate lipid droplets, which may serve as sources for fatty acids or precursors for ketone‐body production, and that BHB itself can suppress microglial pro‐inflammatory activation and preserve mitochondrial function in neurodegenerative or injury contexts [86, 87]. Moreover, inhibition of fatty acid oxidation may trigger compensatory activation of glycolysis, for example, via HIF‐1α, potentially influencing the observed phenotypes. In the current study, markers of glycolysis or metabolic plasticity were not measured; future work should integrate analyses of fatty acid oxidation, glycolysis, and other metabolic pathways to fully characterize microglial metabolism. These represent important directions for future experiments using SNI rat models to further validate and expand these findings.

## 5 Conclusion

All in all, we provide evidence that ChREBP regulates microglial fatty acid oxidation, promotes anti‐inflammatory microglial polarization, suppresses neuroinflammation, reduces the excitability of spinal neurons, and alleviates pain. Mechanistically, ChREBP regulates PGC‐1α expression by binding to its promoter region. Therefore, our study suggests that targeting ChREBP may offer a metabolic‐based therapeutic strategy for alleviating NP.

## Author Contributions

Z.H., J.P., X.L., and Y.Z. contributed equally to this work. Z.H. and J.P. performed the experiments and drafted the manuscript. X.L. and Y.Z. analyzed the data. Y.L., H.C., and H.Z. helped with the manuscript submission. Y.Y., Y.Z., L.G., and X.Z. provided instrumental support. J.J., K.W., and Y.S. assisted in the preparation of the material. H.Z. and W.W. jointly supervised this work.

## Funding

This work was supported by the National Natural Science Foundation of China (82172526, 82372553, and 82402954), Guangdong Basic and Applied Basic Research Foundation (2023A1515010200 and 2021A1515010135), and Guangzhou Municipal Science and Technology Project.

## Ethics Statement

All procedures were approved by the Experimental Animal Ethics Committee of Zhujiang Hospital of Southern Medical University (Ethics No.: LAEC‐2022‐144).

## Conflicts of Interest

The authors declare no conflicts of interest.

## Supporting information


**Figure S1:** Increased expression of ChREBP in the spinal dorsal horn on the ipsilateral side of NP rat models. (A) Representative immunofluorescence staining images of ChREBP expression in the ipsilateral (Ipsi) and contralateral (Contra) spinal dorsal horn after SNI surgery. (B–E) Quantitative analysis of ChREBP fluorescence intensity in lamina I (B), lamina II (C), lamina III (D), and lamina IV (E) on the ipsilateral and contralateral sides. (F) Quantitative analysis of ChREBP fluorescence intensity in the enlarged superficial dorsal horn region. (G) The mRNA levels of ChREBP in the spinal cord on the ipsilateral and contralateral sides after SNI surgery. Data are presented as mean ± SD, *n* = 6, ^ns^ *p* > 0.05, **p < 0.01, ***p < 0.001.
**Figure S2:** Increased expression of ChREBP in HAPI cells exposed to LPS stimulation. (A) The mRNA levels of TNF‐α, IL‐1β, and IL‐6 in HAPI cells of each group were detected by RT‐qPCR. (B) The mRNA level of ChREBP was measured using RT‐qPCR. Data are presented as mean ± SD, *n* = 3, **p < 0.01, ***p < 0.001.
**Figure S3:** ChREBP mediates anti‐inflammatory polarization and inflammation suppression in microglia. HAPI cells exposed to LPS were transfected with siRNA targeting ChREBP and divided into three groups: LPS + siNC (siNC, negative control), LPS + siChREBP‐1, and LPS + siChREBP‐2. (A‐C) RT‐qPCR and western blot analysis of ChREBP mRNA (A) and protein (B, C) expression in each group. (D, E) Representative flow cytometric analysis images (D) and the ratio of CD11b^+^CD86^+^ (pro‐inflammatory microglia) to CD11b^+^CD206^+^ (anti‐inflammatory microglia) cells (E) in each group. (F) mRNA levels of inflammatory cytokines TNF‐α, IL‐1β, and IL‐6 in each group. HAPI cells exposed to LPS were transfected with ChREBP overexpression plasmid and divided into two groups: LPS + EV (EV, empty vector) and LPS + ChREBP. (G–I) RT‐qPCR and western blot analysis of ChREBP mRNA (G) and protein (H, I) expression in each group. (J, K) Representative flow cytometric analysis images (J) and the ratio of CD11b^+^CD86^+^ to CD11b^+^CD206^+^ cells (K) in each group. (L) mRNA levels of inflammatory cytokines TNF‐α, IL‐1β, and IL‐6 in each group.
**Figure S4:** In vivo overexpression of ChREBP increases the number of anti‐inflammatory microglia and alleviates pain. (A) Experimental timeline of SNI surgery, spinal cord stereotaxic injection, and behavioral tests. (B, C) Representative immunofluorescence staining images (B) and quantitative analysis (C) for ChREBP and Iba‐1‐positive cells in the spinal cord. (D) ChREBP mRNA level in the spinal cord. (E) Representative immunofluorescence staining images for double‐labeled Iba‐1 and pro‐inflammatory microglial marker (iNOS) and double‐labeled Iba‐1 and anti‐inflammatory microglial marker (Arg‐1) in the spinal cord. (F–H) Quantitative analysis of immunofluorescence results, including (F) number of Iba‐1^+^iNOS^+^ colocalized cells; (G) number of Iba‐1^+^Arg‐1^+^ colocalized cells; and (H) ratio of Iba‐1^+^iNOS^+^ to Iba‐1^+^Arg‐1^+^ cells. (I, J) Mechanical pain thresholds on the ipsilateral (I) and contralateral (J) sides were measured before microinjection (BFMI), at baseline (BL), and on days 1, 3, 7, 14, and 21 after SNI surgery in rats. Data are presented as mean ± SD, *n* = 6, ^ns^ *p* > 0.05, *p < 0.05, **p < 0.01, ***p < 0.001.
**Figure S5:** Results from the STRING database. (A) A protein–protein interaction (PPI) network associated with ChREBP expression was constructed based on the STRING database, and a total of 50 hub genes were identified. The gene name for ChREBP (the core gene of the network) is MLXIPL, and the gene name for PGC‐1α (a key interacting protein in the network) is PPARGC1A.
**Figure S6:** Results of GO functional annotation. (A) GO enrichment analysis of 50 hub genes significantly associated with ChREBP, categorized into three ontologies: biological process (BP), cellular component (CC), and molecular function (MF).
**Figure S7:** ChREBP regulates fatty acid oxidation and mitochondrial biogenesis proteins through PGC‐1α in microglia. HAPI cells exposed to LPS were transfected with siRNA targeting ChREBP and a PGC‐1α overexpression plasmid and were divided into five groups: Control, LPS + siNC+EV, LPS + siNC+PGC‐1α, LPS + siChREBP+EV, and LPS + siChREBP+PGC‐1α. (A) Representative western blot images showing the protein levels of ChREBP, PGC‐1α, CPT1A, ACADM, NRF1, and TFAM in LPS‐treated microglial cells. (B‐G) Quantification of protein abundance, normalized to β‐actin, for ChREBP (B), PGC‐1α (C), CPT1A (D), ACADM (E), NRF1 (F), and TFAM (G). Data are presented as mean ± SD, *n* = 3, *p < 0.05, **p < 0.01, ***p < 0.001.

**Table S1:** Primer sequences for siRNA targeting ChREBP.
**Table S2:** Primer sequences for each gene.
**Table S3:** The node connection degree of top eight hub genes.

## Data Availability

The data that supports the findings of this study are available in the Supporting Informtion of this article.
