# Supplementary material for: PGC‐1α Transcriptionally Regulated by ChREBP Mitigates Neuropathic Pain Through Promoting Microglial Fatty Acid Oxidation and Anti‐Inflammatory Response
Source: CNS Neurosci Ther. 2026 Jan 10;32(1):e70744. doi: 10.1002/cns.70744 (PMC12789879; doi:10.1002/cns.70744)
Supplement: Supplementary file 1 — Figure S1: Increased expression of ChREBP in the spinal dorsal horn on the ipsilateral side of NP rat models. (A) Representative immunofluorescence staining images of ChREBP expression in the ipsilateral (Ipsi) and contralateral (Contra) spinal dorsal horn after SNI surgery. (B–E) Quantitative analysis of ChREBP fluorescence intensity in lamina I (B), lamina II (C), lamina III (D), and lamina IV (E) on the ipsilateral and contralateral sides. (F) Quantitative analysis of ChREBP fluorescence intensity in the enlarged superficial dorsal horn region. (G) The mRNA levels of ChREBP in the spinal cord on the ipsilateral and contralateral sides after SNI surgery. Data are presented as mean ± SD, n = 6, ns p > 0.05, **p < 0.01, ***p < 0.001. Figure S2: Increased expression of ChREBP in HAPI cells exposed to LPS stimulation. (A) The mRNA levels of TNF‐α, IL‐1β, and IL‐6 in HAPI cells of each group were detected by RT‐qPCR. (B) The mRNA level of ChREBP was measured using RT‐qPCR. Data are presented as mean ± SD, n = 3, **p < 0.01, ***p < 0.001. Figure S3: ChREBP mediates anti‐inflammatory polarization and inflammation suppression in microglia. HAPI cells exposed to LPS were transfected with siRNA targeting ChREBP and divided into three groups: LPS + siNC (siNC, negative control), LPS + siChREBP‐1, and LPS + siChREBP‐2. (A‐C) RT‐qPCR and western blot analysis of ChREBP mRNA (A) and protein (B, C) expression in each group. (D, E) Representative flow cytometric analysis images (D) and the ratio of CD11b+CD86+ (pro‐inflammatory microglia) to CD11b+CD206+ (anti‐inflammatory microglia) cells (E) in each group. (F) mRNA levels of inflammatory cytokines TNF‐α, IL‐1β, and IL‐6 in each group. HAPI cells exposed to LPS were transfected with ChREBP overexpression plasmid and divided into two groups: LPS + EV (EV, empty vector) and LPS + ChREBP. (G–I) RT‐qPCR and western blot analysis of ChREBP mRNA (G) and protein (H, I) expression in each group. (J, K) Representative flow cytometric ana [file CNS-32-e70744-s001.docx]

**Supplementary Materials**

**
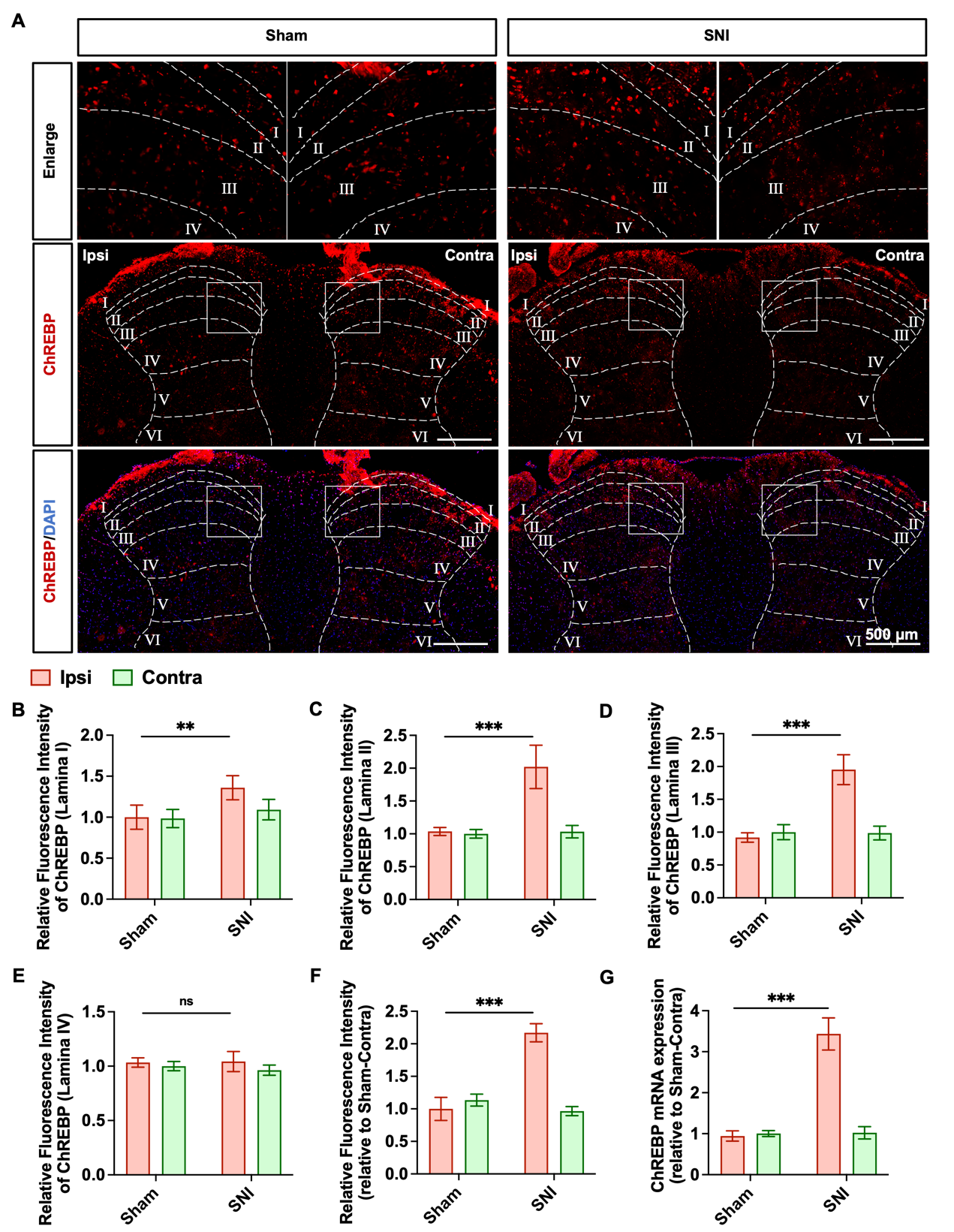
**

**Fig. S1** Increased expression of ChREBP in the spinal dorsal horn on the ipsilateral side of NP rat models. (A) Representative immunofluorescence staining images of ChREBP expression in the ipsilateral (Ipsi) and contralateral (Contra) spinal dorsal horn after SNI surgery. (B–E) Quantitative analysis of ChREBP fluorescence intensity in lamina I (B), lamina II (C), lamina III (D) and lamina IV (E) on the ipsilateral and contralateral sides. (F) Quantitative analysis of ChREBP fluorescence intensity in the enlarged superficial dorsal horn region. (G) The mRNA levels of ChREBP in the spinal cord on the ipsilateral and contralateral sides after SNI surgery. Data are presented as mean ± SD, n = 6, ^ns^ *P* > 0.05, ^**^*P* < 0.01, ^***^*P* < 0.001.


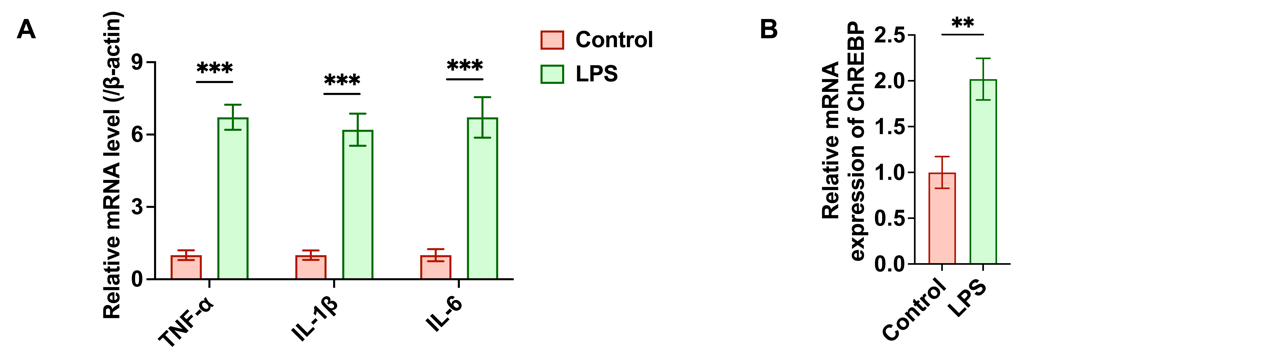


**Fig. S2** Increased expression of ChREBP in HAPI cells exposed to LPS stimulation. (A) The mRNA levels of TNF-α, IL-1β, and IL-6 in HAPI cells of each group were detected by RT-qPCR. (B) The mRNA level of ChREBP was measured using RT-qPCR. Data are presented as mean ± SD, n = 3, ^**^*P* < 0.01, ^***^*P* < 0.001.


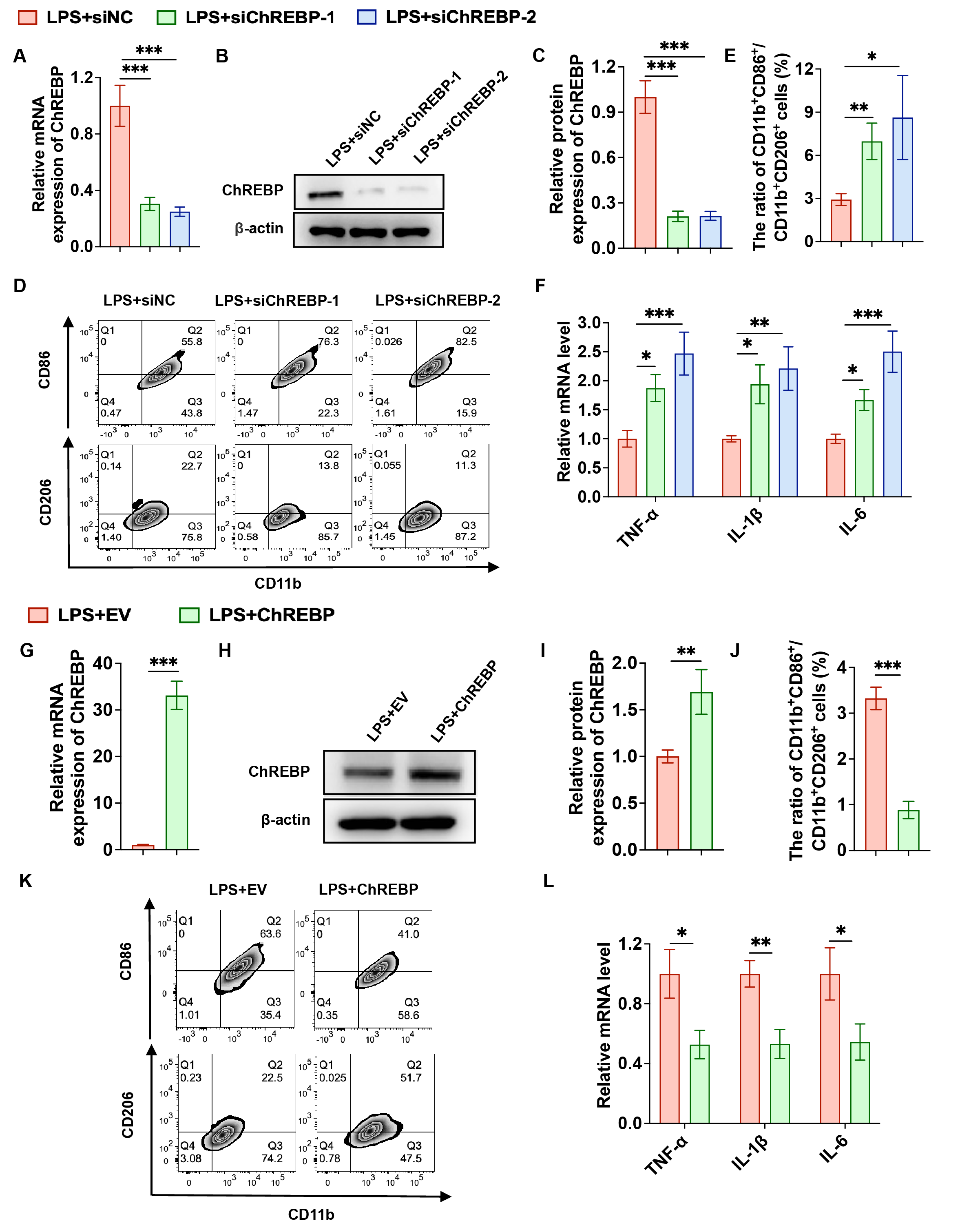


**Fig. S3** ChREBP mediates anti-inflammatory polarization and inflammation suppression in microglia. HAPI cells exposed to LPS were transfected with siRNA targeting ChREBP and divided into three groups: LPS+siNC (siNC, negative control), LPS+siChREBP-1, and LPS+siChREBP-2. (A-C) RT-qPCR and western blot analysis of ChREBP mRNA (A) and protein (B, C) expression in each group. (D, E) Representative flow cytometric analysis images (D) and the ratio of CD11b^+^CD86^+^ (pro-inflammatory microglia) to CD11b^+^CD206^+^ (anti-inflammatory microglia) cells (E) in each group. (F) mRNA levels of inflammatory cytokines TNF-α, IL-1β, and IL-6 in each group. HAPI cells exposed to LPS were transfected with ChREBP overexpression plasmid and divided into two groups: LPS+EV (EV, empty vector) and LPS+ChREBP. (G-I) RT-qPCR and western blot analysis of ChREBP mRNA (G) and protein (H, I) expression in each group. (J, K) Representative flow cytometric analysis images (J) and the ratio of CD11b^+^CD86^+^ to CD11b^+^CD206^+^ cells (K) in each group. (L) mRNA levels of inflammatory cytokines TNF-α, IL-1β, and IL-6 in each group.

Data are represented as mean ± SD, n = 3, ^*^*P* < 0.05, ^**^*P* < 0.01, ^***^*P* < 0.001.

**
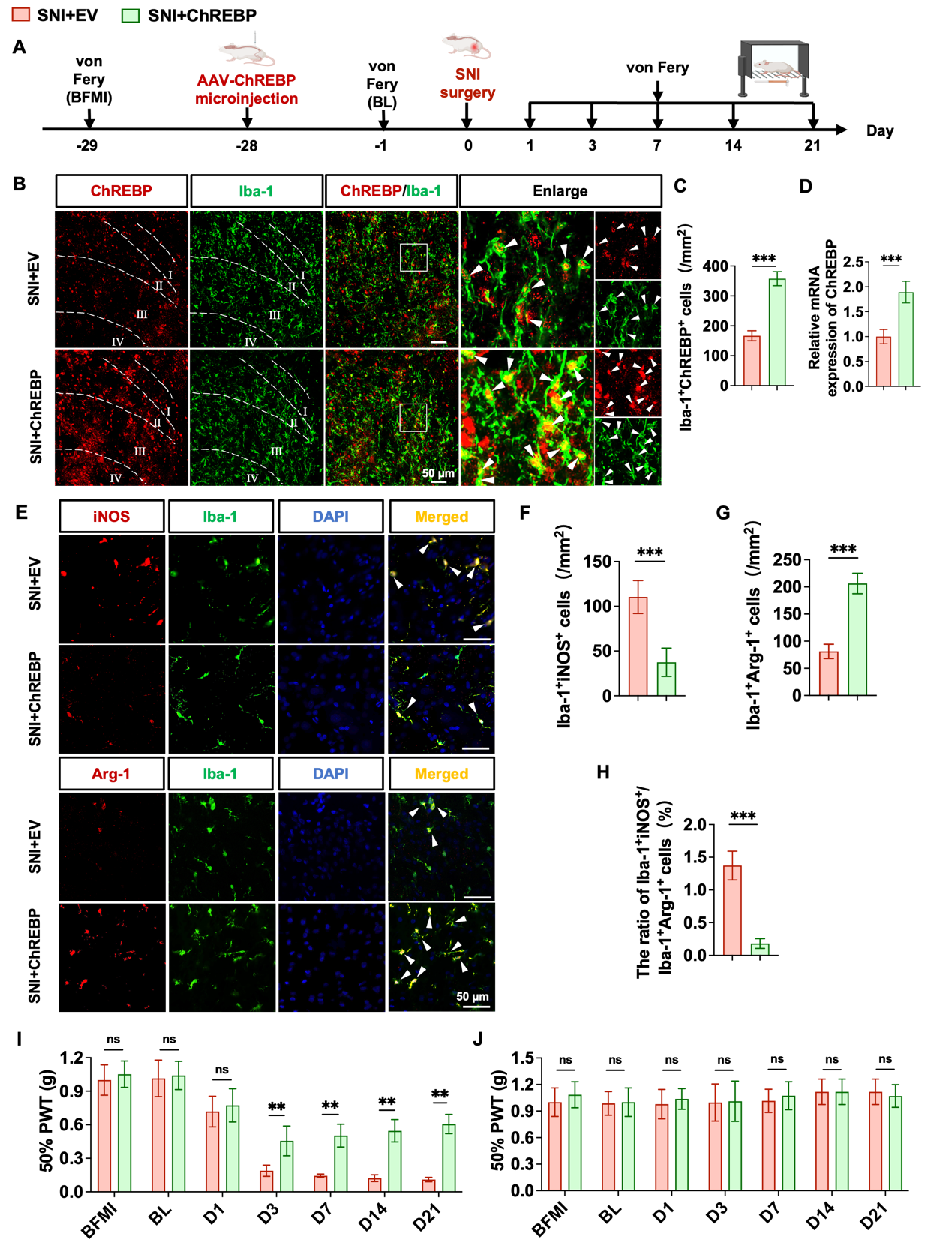
**

**Fig. S4** In vivo overexpression of ChREBP increases the number of anti-inflammatory microglia and alleviates pain. (A) Experimental timeline of SNI surgery, spinal cord stereotaxic injection, and behavioral tests. (B, C) Representative immunofluorescence staining images (B) and quantitative analysis (C) for ChREBP and Iba-1-positive cells in the spinal cord. (D) ChREBP mRNA level in the spinal cord. (E) Representative immunofluorescence staining images for double-labeled Iba-1 and pro-inflammatory microglial marker (iNOS), and double-labeled Iba-1 and anti-inflammatory microglial marker (Arg-1) in the spinal cord. (F–H) Quantitative analysis of immunofluorescence results, including: (F) Number of Iba-1⁺iNOS⁺ colocalized cells; (G) Number of Iba-1⁺Arg-1⁺ colocalized cells; (H) Ratio of Iba-1⁺iNOS⁺ to Iba-1⁺Arg-1⁺ cells. (I-J) Mechanical pain thresholds on the ipsilateral (I) and contralateral (J) sides were measured before microinjection (BFMI), at baseline (BL), and on days 1, 3, 7, 14, and 21 after SNI surgery in rats. Data are presented as mean ± SD, n = 6, ^ns^ *P* > 0.05, ^*^*P* < 0.05, ^**^*P* < 0.01, ^***^*P* < 0.001.


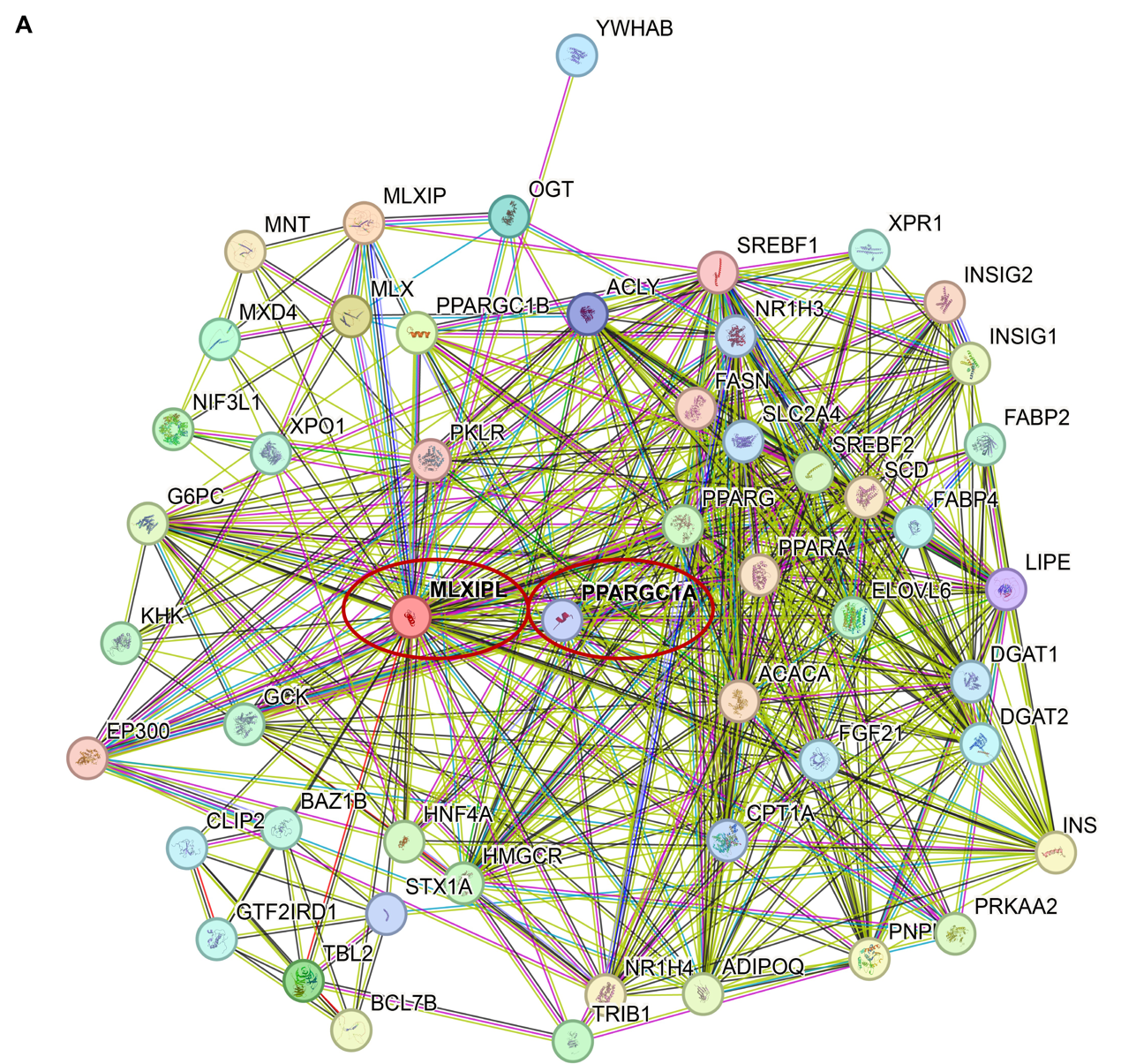


**Fig. S5** Results from the STRING database. (A) A protein-protein interaction (PPI) network associated with ChREBP expression was constructed based on the STRING database, and a total of 50 hub genes were identified. The gene name for ChREBP (the core gene of the network) is MLXIPL, and the gene name for PGC-1α (a key interacting protein in the network) is PPARGC1A.


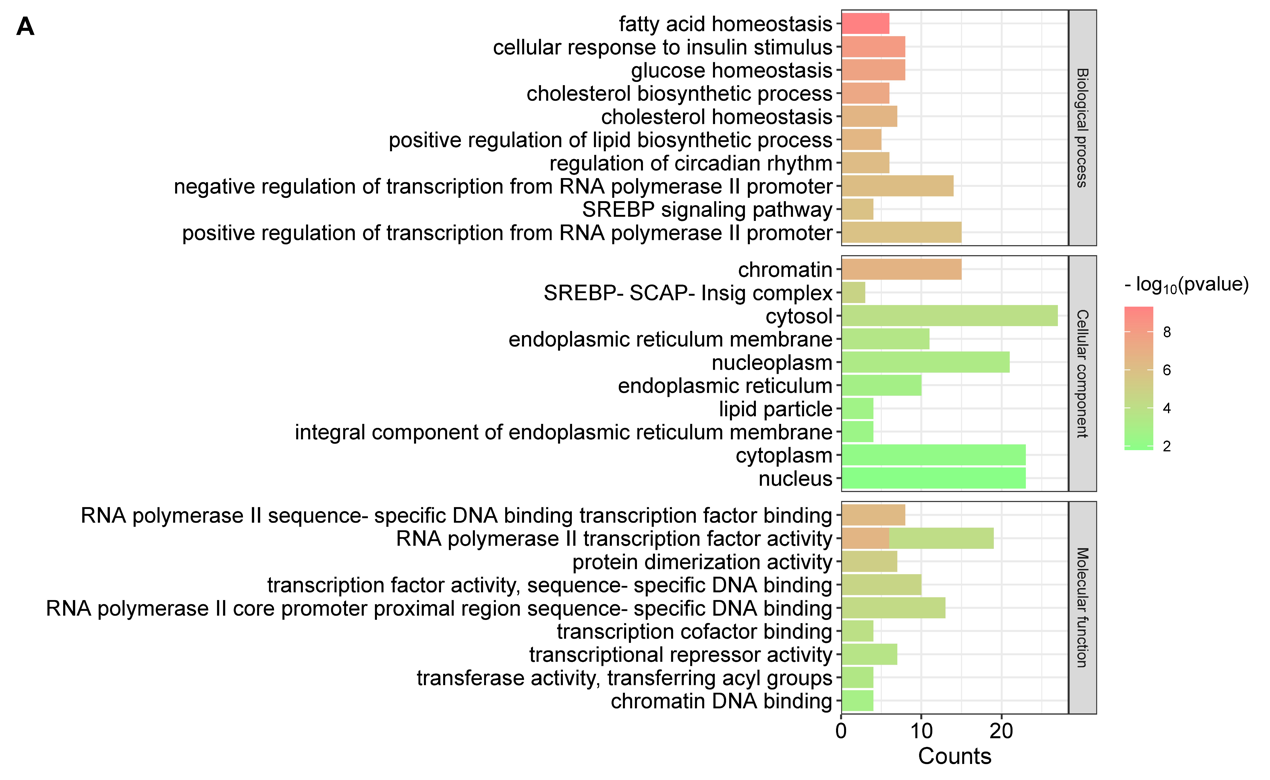


**Fig. S6** Results of GO functional annotation. (A) GO enrichment analysis of 50 hub genes significantly associated with ChREBP, categorized into three ontologies: biological process (BP), cellular component (CC), and molecular function (MF).

**
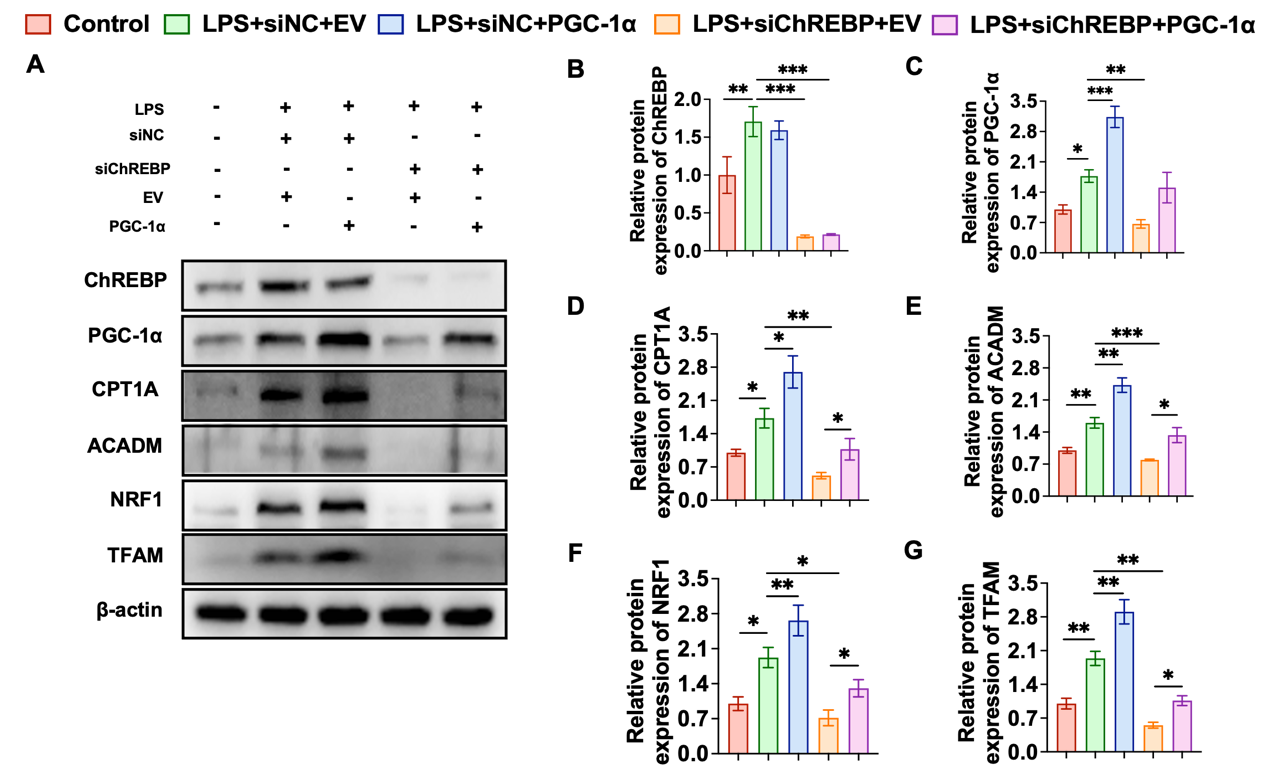
**

**Fig. S7** ChREBP regulates fatty acid oxidation and mitochondrial biogenesis proteins through PGC-1α in microglia. HAPI cells exposed to LPS were transfected with siRNA targeting ChREBP and a PGC-1α overexpression plasmid and were divided into five groups: Control, LPS+siNC+EV, LPS+siNC+PGC-1α, LPS+siChREBP+EV, and LPS+siChREBP+PGC-1α. (A) Representative Western blot images showing the protein levels of ChREBP, PGC-1α, CPT1A, ACADM, NRF1, and TFAM in LPS-treated microglial cells. (B-G) Quantification of protein abundance, normalized to β-actin, for ChREBP (B), PGC-1α (C), CPT1A (D), ACADM (E), NRF1 (F), and TFAM (G). Data are presented as mean ± SD, n = 3, ^*^*P* < 0.05, ^**^*P* < 0.01, ^***^*P* < 0.001.
