# Supplementary material for: PGC‐1α Transcriptionally Regulated by ChREBP Mitigates Neuropathic Pain Through Promoting Microglial Fatty Acid Oxidation and Anti‐Inflammatory Response
Source: CNS Neurosci Ther. 2026 Jan 10;32(1):e70744. doi: 10.1002/cns.70744 (PMC12789879; doi:10.1002/cns.70744)
Supplement: Supplementary file 2 — Table S1: Primer sequences for siRNA targeting ChREBP. Table S2: Primer sequences for each gene. Table S3: The node connection degree of top eight hub genes. [file CNS-32-e70744-s002.docx]

**Table S1** Primer sequences for siRNA targeting ChREBP

| **Name** | **Forward primer (5'-3')** | **Reverse primer (5'-3')** |
| --- | --- | --- |
| siNC | UUCUCCGAACGUGUCACGUTT | ACGUGACACGUUCGGAGAATT |
| siChREBP-1 | CAGAAGAGGCGUUUCAAUATT | UAUUGAAACGCCUCUUCUGTT |
| siChREBP-2 | GGGACAUGUUUGAUGACUATT | UAGUCAUCAAACAUGUCCCTT |

**Table S2** Primer sequences for each gene

| **Gene name** | **Forward primer (5'-3')** | **Reverse primer (5'-3')** |
| --- | --- | --- |
| ChREBP | GTACTGTTCCCTGCCTGCTCTC | CCCTCTGTGACTGCCCTTGTG |
| TNF-α | CCGAGATGTGGAACTGGCAGAG | CCGCCACGAGCAGGAATGAG |
| IL-1β | ACAGCAGCATCTCGACAAGAGC | CCACGGGCAAGACATAGGTAGC |
| IL-6 | AGACTTCCAGCCAGTTGCCTTC | AAGCCTCCGACTTGTGAAGTGG |
| PPARG | CTGTGGACCTCTCTGTGATGGATG | AACGGGATGTCTTCATAGTGTGGAG |
| SREBF1 | CTTACAGCACAGCAACCAGAAACTC | GCCTCCTCCACTGCCACAAG |
| SCD | TCGTCAGCACCTTCTTGAGATACAC | GCACCCAGGGAAACCAGGATG |
| FASN | TGTGGTAGGCTTGGTGAACTGTC | TGAGATGTGCTGCTGAGGTTGG |
| PGC-1α | CATGCAAACCACACCCACAG | CTGAGCAGGGACGTCTTTGT |
| ACACA | CGATTCCCATCCGCCTCTTCC | GGTCCCTGCTTGTCTCCATACG |
| PPARA | TCTTCACGATGCTGTCCTCCTTG | TGTCGCAGAATGGCTTCCTCAG |
| HMGCR | CGCAACCTCTACATCCGTCTCC | ATAGTTACCACTGACCGCCAGAATC |
| CPT1A | AAGTCAACGGCAGAGCAGAGG | GGACACCACATAGAGGCAGAAGAG |
| CPT2 | AATGACCAGCTTACCAGGGC | TCTAAAAGGCCAGCCCGAAG |
| ACADM | GAGGCTACAAGGTCCTGAGAAGTG | TTCTGCTGCTCCGTCAACTCG |
| HADHA | CAGAGCAAAGGCCTGATGGA | GTCCAGTCTTCACAGCGTCA |
| β actin | TGAGAGGGAAATCGTGCGTGAC | GGAAGAGGATGCGGCAGTGG |

**Table S3** The node connection degree of top 8 hub genes.

| **Number** | **Gene name** | **Degree** |
| --- | --- | --- |
| 1 | PPARG | 35 |
| 2 | SREBF1 | 34 |
| 3 | SCD | 31 |
| 4 | FASN | 31 |
| 5 | PGC-1α | 31 |
| 6 | ACACA | 31 |
| 7 | PPARA | 30 |
| 8 | HMGCR | 30 |
